# MYC-driven increases in mitochondrial DNA copy number occur early and persist throughout prostatic cancer progression

**DOI:** 10.1172/jci.insight.169868

**Published:** 2023-12-22

**Authors:** Jiayu Chen, Qizhi Zheng, Jessica L. Hicks, Levent Trabzonlu, Busra Ozbek, Tracy Jones, Ajay M. Vaghasia, Tatianna C. Larman, Rulin Wang, Mark C. Markowski, Sam R. Denmeade, Kenneth J. Pienta, Ralph H. Hruban, Emmanuel S. Antonarakis, Anuj Gupta, Chi V. Dang, Srinivasan Yegnasubramanian, Angelo M. De Marzo

**Affiliations:** 1 Department of Pathology, Johns Hopkins University School of Medicine, Baltimore, Maryland, USA.; 2 Department of Oncology and; 3 Department of Urology, Johns Hopkins University School of Medicine, Baltimore, Maryland, USA.; 4 The Sol Goldman Pancreatic Cancer Research Center, Johns Hopkins Medicine, Baltimore, Maryland, USA.; 5 Department of Biochemistry and Molecular Biology, Johns Hopkins Bloomberg School of Public Health, Baltimore, Maryland, USA.

**Keywords:** Metabolism, Oncology, Cancer, Mitochondria

## Abstract

Increased mitochondrial function may render some cancers vulnerable to mitochondrial inhibitors. Since mitochondrial function is regulated partly by mitochondrial DNA copy number (mtDNAcn), accurate measurements of mtDNAcn could help reveal which cancers are driven by increased mitochondrial function and may be candidates for mitochondrial inhibition. However, prior studies have employed bulk macrodissections that fail to account for cell type–specific or tumor cell heterogeneity in mtDNAcn. These studies have often produced unclear results, particularly in prostate cancer. Herein, we developed a multiplex in situ method to spatially quantify cell type–specific mtDNAcn. We show that mtDNAcn is increased in luminal cells of high-grade prostatic intraepithelial neoplasia (HGPIN), is increased in prostatic adenocarcinomas (PCa), and is further elevated in metastatic castration-resistant prostate cancer. Increased PCa mtDNAcn was validated by 2 orthogonal methods and is accompanied by increases in mtRNAs and enzymatic activity. Mechanistically, MYC inhibition in prostate cancer cells decreases mtDNA replication and expression of several mtDNA replication genes, and MYC activation in the mouse prostate leads to increased mtDNA levels in the neoplastic prostate cells. Our in situ approach also revealed elevated mtDNAcn in precancerous lesions of the pancreas and colon/rectum, demonstrating generalization across cancer types using clinical tissue samples.

## Introduction

Otto Warburg observed in the 1920s that cancer cells take up excess glucose that is metabolized mostly to lactate, even in the presence of oxygen. He postulated that such aerobic glycolysis (the Warburg effect) was universal in cancer, essential for cancer cell proliferation, and caused by disrupted mitochondrial respiration (1). The concept of mitochondrial dysfunction as oncogenic was later supported by studies showing that a number of cancer types contain somatic point mutations and/or deletions in mitochondrial DNA (mtDNA) (2–5). In addition, inactivating mutations in nuclear genes whose protein products function in mitochondria occur in several hereditary cancer syndromes, including those caused by mutations in fumarate hydratase (FH) and succinate dehydrogenase (SDH) (6–9). In the prostate, somatic mutations in mtDNA have been proposed as potential cancer drivers (10–18). Furthermore, a 3.4 kb deletion in mtDNA has been reported in prostate cancer, and these and other mtDNA deletions accumulate in the aging prostate (16, 17, 19). Nevertheless, the functional significance of somatic mtDNA alterations as oncogenic drivers in cancer, including prostate cancer, remains to be fully clarified.

The phenomenon of aerobic glycolysis, which is the use of glycolysis rather than oxidative phosphorylation as a source of energy in cells even in aerobic conditions, was long proposed as a unique property of cancer cells indicative of mitochondrial dysfunction in cancer (20, 21). However, recent evidence has shown that such aerobic glycolysis is not cancer specific and commonly occurs in nonneoplastic proliferating cells as part of a metabolic program during cell growth and division (1, 20–26). While a complete picture of precisely why dividing cells undergo aerobic glycolysis is still emerging, the metabolism of glucose to lactate helps maintain high levels of glycolytic intermediates and/or NAD^+^ to support anabolic reactions needed for cell growth and replication (1, 21, 22, 24, 27). Furthermore, multiple lines of evidence suggest that cancer and other nonneoplastic proliferating cells have increased mitochondrial activity. Several metabolites generated by the TCA cycle are used as intermediates for macromolecular synthesis in proliferating cells. Also, except for a relatively rare subset of cancer types (e.g., oncocytomas of the kidney, renal tumors associated with inactivation of *FH*, or genes encoding the SDH complex), respiration is not generally markedly impaired in cancer (21, 25, 28), and a functioning electron transport chain is required for proliferation of most normal and cancer cells (21, 29–32). Taken together, it is now apparent that mitochondria display increased activity in most cancers; however, their metabolic activities are reprogrammed to facilitate cell proliferation and continuous self-renewal (1, 2, 21, 22, 24–28, 33).

Mitochondrial function is regulated by mitochondrial biogenesis, which is controlled, at least partially, by mtDNA copy number (mtDNAcn) (34, 35). Alterations in mtDNAcn have been reported in cancer (35). For example, increased mtDNAcn has been described in acute myelogenous leukemia (AML) (36, 37), endometrial carcinoma (38), esophageal squamous cancer (39), pancreatic cancer (5), and colorectal cancer (40). Furthermore, treatment with an inhibitor of mtDNA replication (41) or of a mitochondrial Complex I inhibitor (42) in AML has shown promise in preclinical studies. Notably, increased mtDNA content was mechanistically linked to microsatellite-stable colorectal cancer cell proliferation and metastasis (40). However, in other cancers, such as gastric, breast, hepatocellular, non–small cell lung, and renal carcinomas, decreased mtDNA levels — as compared with their normal tissue counterparts — have been reported (35). Thus, there is no general pattern regarding mtDNAcn changes in tumor-normal comparisons in common adult cancer types.

In terms of mtDNAcn changes in prostate cancer, there are contradictory results (16, 43–45). Mizumachi et al. examined single laser microdissected cells from a small number (*n* = 9) of prostate cancers and reported increased mtDNA content in some cancers, decreased amounts in others, and a general increase in cell-to-cell variability in cancer cells compared with matched benign tissues (43). Koochekpour et al. reported lower levels of mtDNA in human prostate cancer compared with normal prostate tissues and suggested that reduced levels of mtDNA can drive aggressive prostate cancer (44). Reznik et al. analyzed mtDNAcn variation across multiple cancers relative to matched nonneoplastic tissues using next-generation sequencing (NGS) data from The Cancer Genome Atlas (TCGA), finding that prostate cancer had nonstatistically significantly increased bulk tumor mtDNA levels compared with benign prostate tissues (45). Schöpf et al. reported that there was no significant difference in mtDNAcn between benign and malignant prostate tissues, although mtDNAcn was increased in higher-grade cancers (18). Higuchi et al. showed that androgen-independent C4-2B cells had lower levels of mtDNA than androgen-sensitive LNCaP cells and that depletion of mtDNA in LNCaP cells resulted in the development of androgen independence (46). Consequently, whether there are increased or decreased levels of mtDNA in prostate cancer, and whether mtDNA levels are associated with disease aggressiveness and castration resistance, remains unclear. To improve our understanding of the role of mtDNAcn alterations in prostate cancer, it is important to resolve these inconsistent findings.

A concern with most prior studies of mtDNAcn alterations in cancer is that they usually employ bulk macrodissection of tumor and matched benign tissues, which is followed by cellular disruption and DNA isolation. These bulk mtDNA measurements could not take into account heterogeneity within or between tumor nodules or the diversity of cell types (47). We have shown, using an in situ hybridization (ISH) approach, differing mtDNA levels in normal tissue compartments and cell types in most organs (48). Therefore, the use of bulk macrodissections may be ill suited for tumor/normal comparisons since they do not accurately represent differences among relevant neoplastic and nonneoplastic cell types or regional heterogeneity in mtDNA levels of neoplastic cells (43). To better understand the potential biological role of mtDNAcn changes in cancer initiation and progression, it is critical to determine when during the carcinogenesis process the alterations occur (49). Since cancer precursor lesions are often difficult to obtain from fresh tissues and are often microscopic in size, most studies of mtDNAcn alterations on such precursors require either laser-capture microdissection (LCM) or an in situ approach (49).

Here, we report the enhancement of our in situ approach to mtDNA quantification (48) by combining it with iterative sequential IHC in a multiplex assay, allowing for cell type– and compartment-specific mtDNA measurements. This multiplex assay revealed generally increased mtDNAcn in preneoplastic lesions and prostate cancer. Because we and others have linked MYC overexpression to prostate cancer development and progression (50–52), and because MYC has been shown to induce mitochondrial biogenesis (53), we also investigated the role of MYC in regulating mtDNAcn in human and murine prostate cancer cells. These studies revealed that MYC increases mtDNAcn through stimulating mtDNA replication, apparently by increasing expression of mtDNA replication factors, including the helicase TWINKLE.

## Results

### Basal cells show higher mtDNAcn than luminal cells in benign prostate glands.

**Figure 1:**
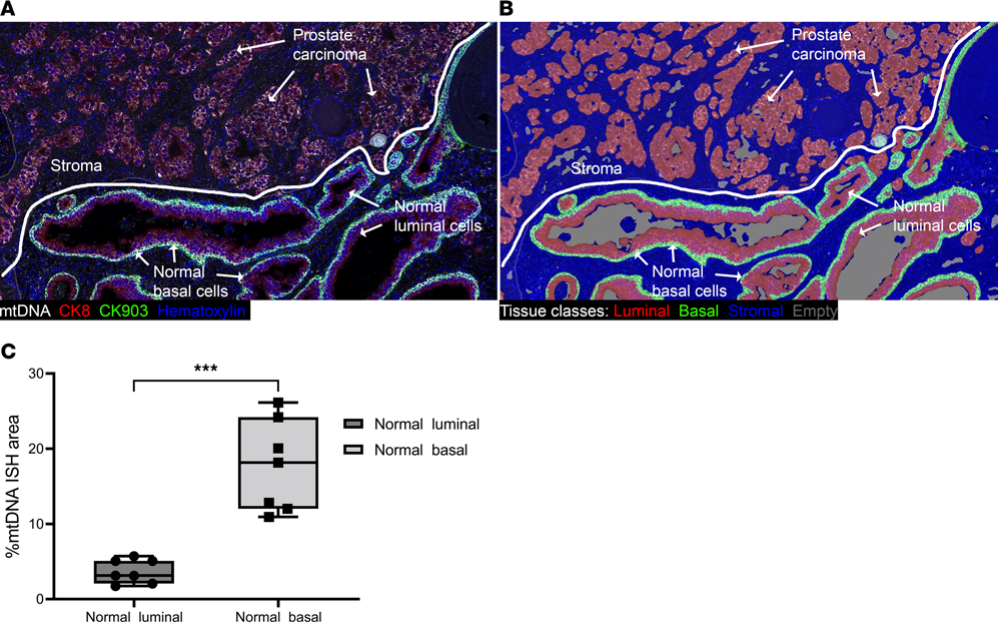
Multiplex chromogenic ISH for mtDNA and IHC in human prostate. (**A**) Whole slide scans of each staining round were imported into HALO and registered, followed by color deconvolution, scan fusions and application of pseudocolors to the resultant channels. mtDNA ISH signals are shown in white, epithelial cells (both basal and luminal) stained with CK8 are shown in red, basal cells stained with CK903 are shown in green, and nuclei are shown in blue (hematoxylin channel). (**B**) Markup image of classifier result showing basal cells (green, present in normal), luminal cells (red, present in normal and at all stages of prostate cancer tumorigenesis), stroma (blue), and empty space/lumens (gray). (**C**) Image analysis results showing higher mtDNAcn in normal basal cells compared with normal luminal cells. The center line in the box shows the median %mtDNA area in each group. *n* = 7 regions from 3 patients for each cell type. Mann-Whitney *U* test, ***P < 0.001. Total original magnification, ×120.

Using chromogenic ISH (CISH), we previously observed that mtDNA signals were visually higher in prostate basal cells compared with normal luminal cells (48). To quantitatively assess mtDNAcn in tissues, we combined mtDNA CISH with multiplex IHC using 2 protein markers, keratin 8 (CK8) and a combination of keratins 5+14 (CK903). Anti-CK8 labels both basal and luminal prostate epithelial cells, and — in normal (e.g., nonatrophic) epithelium — antikeratins 5+14 labels only prostate basal cells. We employed an iterative chromogenic approach (54) in which we first performed ISH for mtDNA followed by whole slide scanning and sequential IHC for each of the keratin antibodies (Figure 1, A and B). We used radical prostatectomy specimens to compare regions containing normal-appearing epithelium, high-grade prostatic intraepithelial neoplasia (HGPIN) and adenocarcinoma. Figure 1A shows the cellular compartmental segmentations from a representative case containing adjacent normal-appearing epithelium and invasive adenocarcinoma. Quantitative image analysis demonstrated that basal cells contained an approximately 5-fold higher mtDNA percent signal area on average than normal-appearing luminal cells (Figure 1C). To address whether the increased mtDNAcn in basal cells compared with luminal cells might be a response to nearby cancer, we studied prostate samples from young adult male organ donors without cancer, and the results were highly similar to those obtained using the samples from patients with cancer (Supplemental Figure 1; supplemental material available online with this article; https://doi.org/10.1172/jci.insight.169868DS1).

### Increased mtDNAcn in HGPIN.

**Figure 2:**
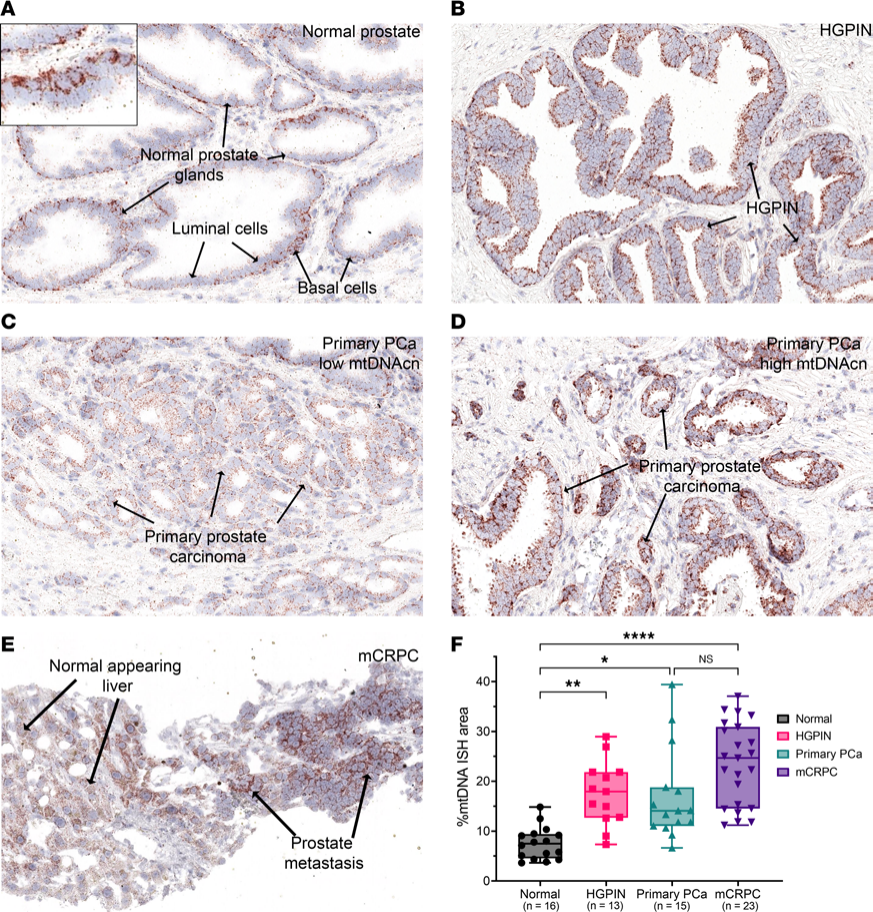
Chromogenic ISH for mtDNA from prostate samples at different stages of prostatic tumorigenesis and image analysis results. Tissues were subjected to multiplex CISH-IHC as in Figure 1, and images from the round of mtDNA ISH are shown. (**A**–**E**) Normal-appearing prostate glands (inset showing higher hybridization signals in normal basal cells) (**A**), HGPIN (**B**), invasive adenocarcinoma with low mtDNA CISH signals (**C**), invasive adenocarcinoma with high mtDNA CISH signals (**D**), and metastatic prostate adenocarcinoma to the liver (**E**). (**F**) Quantification of mtDNA in epithelium presented as %mtDNA area at each stage. Each symbol in the graph is the average of %mtDNA area from multiple regions in a single patient. *n* = 16, 13, 15, and 23 patients for each lesion type. Kruskal-Wallis test followed by Dunn’s test for nonparametric pairwise multiple comparisons. Normal versus HGPIN, **P = 0.0039; normal versus primary PCa, *P = 0.0125; normal versus mCRPC, ****P < 0.0001; and primary PCa versus mCRPC, *P* = 0.1005. Note that images shown in **A**–**D** were from the same patient. Total original magnification, ×200 (**A**–**E**), ×400 (inset in **A**).

HGPIN is the likely precursor to most human prostate cancers (55, 56), yet mtDNAcn levels have not been studied in these lesions. We sought to compare neoplastic epithelial cells in HGPIN to matched benign normal-appearing epithelium. By visual inspection, luminal cells in HGPIN showed increased mtDNA signals as compared with normal luminal cells (Figure 2, A and B). By image analysis, HGPIN showed a consistent increase in the median percent mtDNA signal area of approximately 3-fold compared with the normal prostate glands (Figure 2F). This increase was found despite the fact that basal cells were not excluded from the image analysis in the normal regions.

### Increased overall and heterogeneously distributed mtDNAcn in primary prostate cancer.

Using the same specimens, we next examined primary invasive adenocarcinomas. Visually, invasive carcinomas generally showed higher mtDNAcn than matched normal regions. Interestingly, there was considerable regional heterogeneity of mtDNA ISH signals, both within individual tumor foci (intratumorally) and between foci separated in space (intertumorally) (Figure 2, C and D, and Supplemental Figure 3). Quantitative analysis including the different subregions of tumor foci is shown in Supplemental Figure 2. As an example of intratumoral heterogeneity in a primary prostatic adenocarcinoma, Supplemental Figure 3 shows striking mtDNAcn heterogeneity within an individual tumor from a prostatectomy case. In addition to being contiguous in space, this tumor showed homogeneous ERG expression, indicative of a *TMPRSS2-ERG* gene rearrangement (57, 58). While additional analyses would be needed to definitively assess clonality, this homogeneous ERG expression is consistent with the likelihood that this large tumor with heterogeneous mtDNAcn represents a single clonal origin (Supplemental Figure 3). Taken together, carcinoma lesions had significantly higher levels than normal epithelium and were similar to HGPIN (Figure 2F).

**Figure 3:**
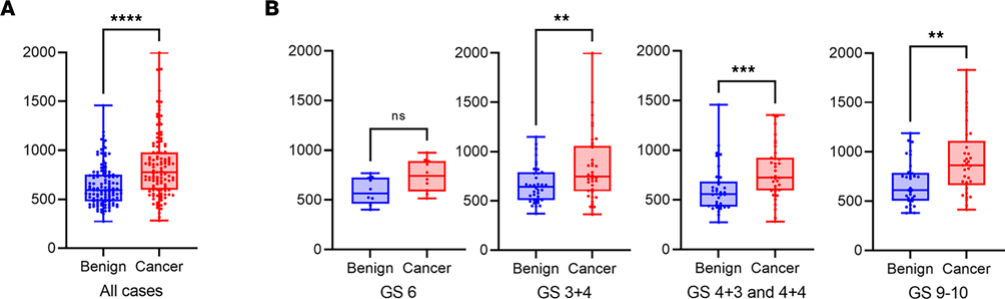
Increased mtDNAcn in prostate cancer using whole-genome sequencing. (**A**) Combined dot and box-and-whisker plot showing increased mtDNA in tumor samples. (**B**) Increased mtDNAcn was present across all Gleason Grade groups (GG) (Gleason Score [GS] 3+3 = GG1; GS 3+4 = GG2; GS 4+3 = GG3 and 4+4 = GG4; GS9-10 = GG5). *n* = 10, 36, 35, and 33 for GS 6, GS 3+4, GS 4+3 and 4+4, and GS 9-10, respectively. Note that GG3 and GG4 are combined due to the relatively low number of GG4 (4+4 = 8) cases. For benign versus cancer, Wilcoxon test was used. All cases, ****P < 0.0001; GS6, *P* = 0.0645; GS 3+4, **P = 0.0013; GS 4+3 and 4+4, ***P = 0.0005; GS 9-10, **P = 0.0024. Blue dots represent benign samples, and red dots represent cancer samples.

As an independent method for comparing mtDNAcn in prostatic tumor-normal pairs, we used whole genome sequencing from laser-capture microdissected tissues in which tumor regions and matched benign paired prostatic regions (non-HGPIN containing) were isolated separately (*n* = 115 prostate cancer cases). In this cohort, which is part of a comprehensive genomic analysis of prostatectomy samples, tumor samples showed increased mtDNAcn compared with matched normal-appearing glands (Figure 3A). Figure 3B shows that the increased mtDNA levels in carcinomas occurred across all major Gleason Grade groups. The distribution of cases by grade, pathological stage, race, and other demographic features are shown in Supplemental Table 2. While there was no clear increase in mtDNAcn by grade, all of the cancers with mtDNAcn signals above 1,000 were Gleason Grade group 2 or higher (Supplemental Table 3).

A prior study has reported that tumor and benign prostate tissues contain differing levels of mtDNAcn in Black compared with White patients (44). When comparing mtDNAcn in tumor samples between White and Black men (self-identified race) using our NGS approach, there were highly similar mtDNAcn levels between them, with no statistical difference (Supplemental Figure 4A).

Aliquots of the same DNA samples used for WGS were subjected to quantitative PCR (qPCR), as described previously (48). This showed a strong positive correlation between the mtDNAcn obtained using WGS and qPCR (Supplemental Figure 4B), validating the quantitative nature of our NGS approach. Also, qPCR on a subset of the samples used for WGS also showed an increase in mtDNAcn in matched tumor samples compared with benign (Supplemental Figure 4C). Thus, 3 independent/orthogonal methods used in this study all show increased mtDNAcn in tumors versus normal prostatic tissues. We observed a weak positive correlation between age and mtDNAcn in the tumor samples, although not in the benign ones (Supplemental Figure 4D) (15). When examining the results by self-reported race, the same trends were present in both Black and White patient samples, although this correlation between age and mtDNAcn in tumor tissue reached statistical significance only in the samples from White patients (data not shown).

### Metastatic castration-resistant prostate cancer showed higher mtDNAcn with less heterogeneity than the primary prostate cancer.

We performed the same iterative CISH-IHC and image analysis workflow using tissues from men with known metastatic castration-resistant prostate cancer (mCRPC) (*n* = 23 patients) using pretreatment biopsies of patients in 2 different clinical trials (51, 59). Visually, these metastases demonstrated high mtDNA ISH signals compared with the adjacent nonneoplastic tissues (when present), with some cases showing higher mtDNA signals than hepatocytes in liver, which are known for high mtDNA content (Figure 2E) (48). By image analysis, we observed high mtDNAcn in most of these mCRPC biopsies, which appeared higher as a group when compared with the primary tumors (Figure 2F).

### Functional relevance of increased mtDNAcn in prostate cancer.

**Figure 4:**
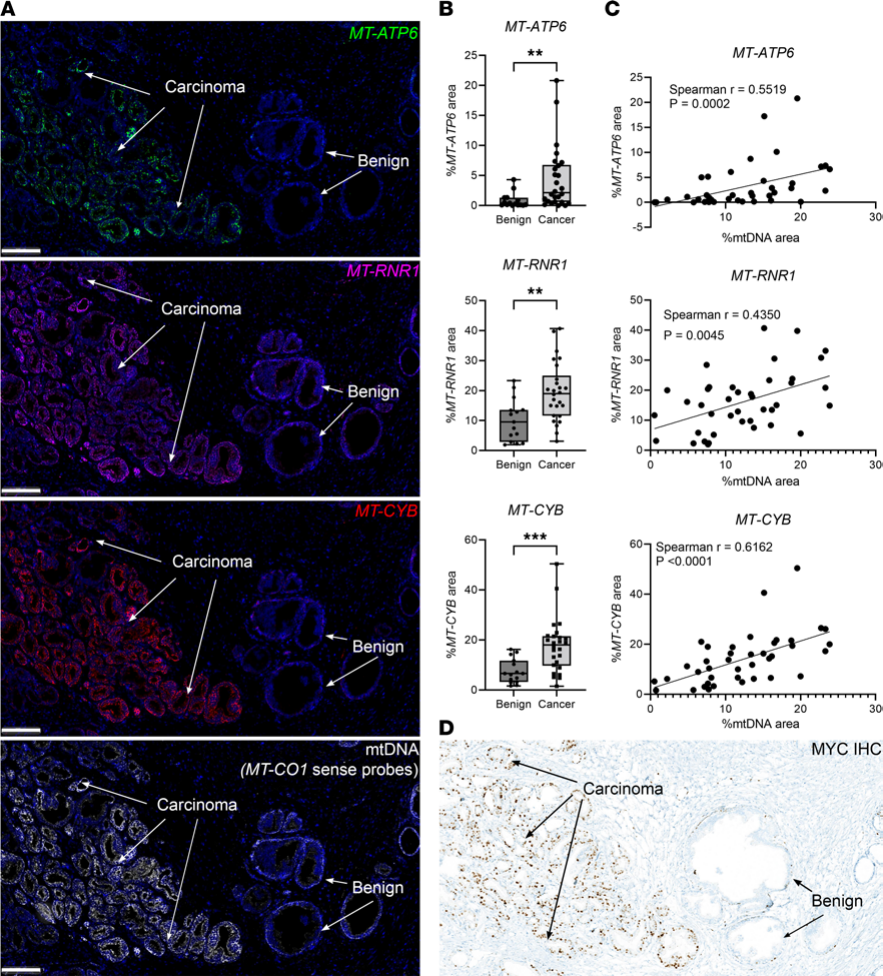
Increased mtDNAcn was accompanied by elevated mtRNA expression as well as MYC protein expression in the prostate. (**A**) Representative images of simultaneous multiplex detection and visualization of mtDNA and mtRNA. (**B**) Image analysis results of 3 mtRNA expression in cancer regions compared with the adjacent benign tissues. Each symbol represents an analyzed region of interest. For benign versus cancer, Mann-Whitney *U* test was used; *MT-ATP6*, **P = 0.0019; *MT-RNR1*, **P = 0.0021; *MT-CYB*, ***P = 0.0005. (**C**) Correlations between mtDNAcn and mtRNA expression by image analysis. (**D**) IHC for MYC from an adjacent frozen section of prostate tissue from the same tissue shown in **A**. Total original magnification, ×70. Scale bar: 200 μm (**A**). *n* = 6 patients.

To address the functional significance of mtDNA alterations in prostate lesions, we first sought to determine whether the increased mtDNAcn was accompanied by corresponding increases in mtRNA. We used a multiplex fluorescence ISH assay to perform simultaneous hybridizations for mtDNA along with mtRNAs from 3 different mtDNA-encoded genes. In the normal-appearing prostate regions, we found generally increased signals in the basal versus luminal compartments, and in carcinoma tissues, there were increased levels for all 3 of these mtRNAs (Figure 4, A and B). Furthermore, there was a correlation between each of these 3 mtRNA species and mtDNA (*MT-CO1* sense) (Figure 4C). In fact, the mtDNA levels correlate with the levels of both MT-rRNA and MT-mRNAs that it encodes. These observations suggest that mtRNA levels may be regulated, at least in part, by mtDNA levels. In addition, we performed enzyme histochemistry for cytochrome coxidase (COX) and SDH on frozen sections from a number of cases (*n* = 12 tissue blocks from 11 patients). In general, there were visually higher levels of both enzyme activities in basal cells compared with luminal cells (Supplemental Figure 5). In prostate carcinomas, there were visually apparent increases as well as heterogeneity that were similar in pattern to the mtDNA in situ signals from adjacent tissue sections, providing further support for the hypothesis that the increased mtDNAcn is functional with respect to production of the encoded genes. Interestingly, the lesions with high mtDNAcn, mtRNA expression, and mitochondrial enzymatic activities also showed high MYC protein expression, suggesting a mechanistic link between them (Figure 4D).

### MYC as a driver of mtDNAcn in clinical prostate cancer.

**Figure 5:**
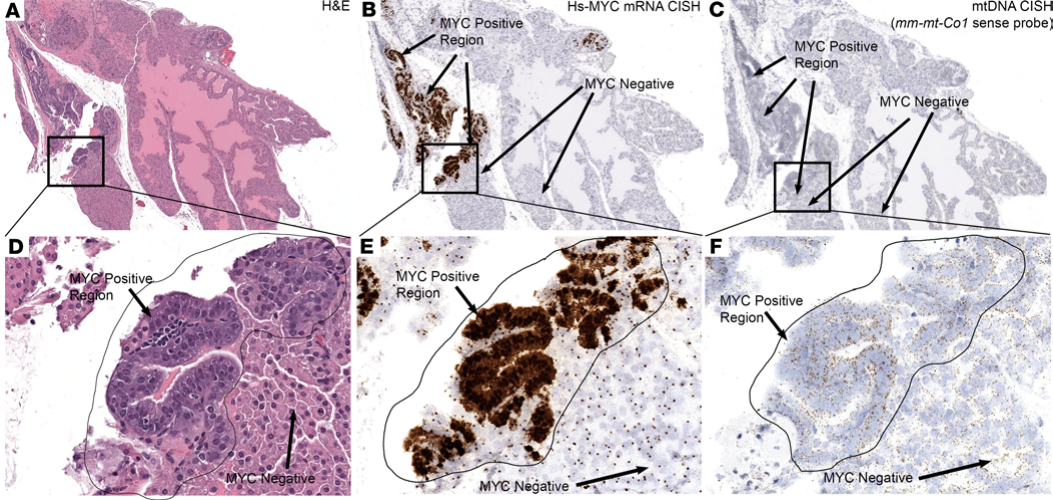
Mouse mtDNAcn is increased in prostates of the Hi-MYC mouse model. (**A** and **D**) H&E images showed the enlarged nuclei and nucleoli, as well as cytoplasmic hyperchromasia characteristic of the mouse PIN lesions after MYC overexpression. (**B**, **C**, **E**, and **F**) Through chromogenic ISH, human MYC mRNA (**B** and **E**) and mtDNA levels (**C** and **F**) were visualized (positive signals are brown). Note the tight spatial correlation with the appearance of cytoplasmic *MYC* signals, corresponding to human *MYC* mRNA expression, and increased mtDNA signals. Also note that the *MYC* probe hybridizes to all nuclei in the transgenic mice as a result of binding to the transgene present in all of the Hi-MYC mouse cells. Total original magnification, ×70 (**A**–**C**), ×200 (**D**–**F**). *n* = 5 mice.

To begin to assess the molecular mechanism for increased mtDNA levels in HGPIN and PCa, we focused on MYC, since MYC has been shown to regulate mitochondrial biogenesis and mtDNA levels (53, 60). MYC is also a key driver of prostate cancer, and its overexpression starts early in the disease process in HGPIN (56, 61, 62), continues in primary carcinomas, and is also highly expressed in mCRPC (52, 61–63). In human prostate cancer cell lines, we have shown that forced overexpression of MYC results in increased mtDNA (48). Despite these findings, since some MYC-driven processes are context- and cell type–specific, including its effects on mitochondrial-related gene expression (53, 60), it is important to investigate whether MYC could be a driver of mtDNAcn changes in normal prostatic epithelial cells in vivo in the context of intact tissues. Therefore, we examined mice with targeted MYC overexpression in the prostate in which MYC is known to drive the development of prostatic intraepithelial neoplasia (PIN) lesions (Hi-MYC mice) (64–66). We focused on the anterior prostate lobe of Hi-MYC, since, despite being driven by AR signaling, MYC expression is quite heterogeneous and it often contains regions of MYC^+^ and MYC^–^ negative epithelium within the same lobe. We observed that, in regions showing increased MYC mRNA and protein levels, there were increased levels of mouse mtDNA that corresponded to the regions of MYC overexpression and accompanying morphological changes diagnostic of PIN, including marked nucleolar enlargement (4 of 4 regions in 3 mice). Figure 5 shows a representative case in a Hi-MYC mouse.

### Decreased mtDNAcn in response to high-dose testosterone treatment in mCRPC and correlation with MYC.

**Figure 6:**
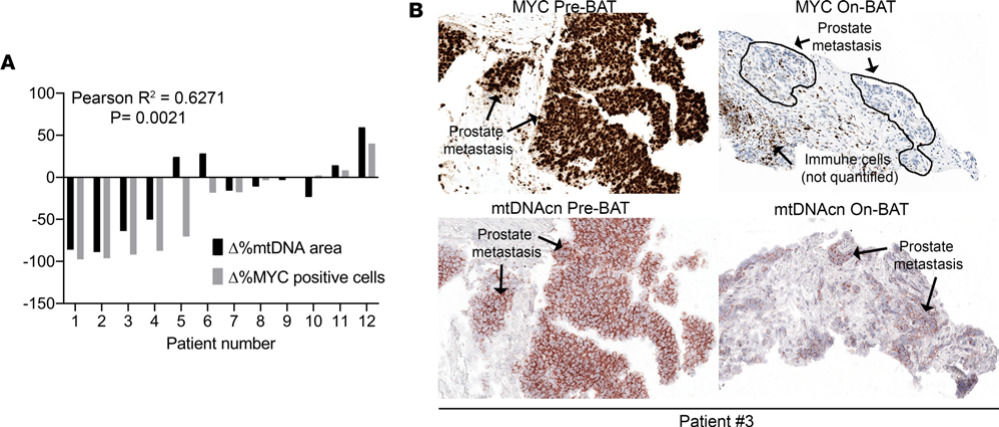
The decrease in MYC in response to SPA correlates with decreased mtDNAcn in mCRPC. (**A**) Each pair of bars shows the percentage change from the baseline biopsy in the percentage of MYC^+^ cells (by IHC staining for MYC protein) among all epithelial tumor cells by image analysis to the percentage change in mtDNA area by image analysis. There was a highly significant correlation (*P* = 0.0021, *R*^2^ = 0.63). (**B**) Representative images (patient no. 3) of MYC-IHC and mtDNAcn-ISH for patients at the pretreatment time point and after 3 cycles of BAT. Notice that, with both markers, image analysis was only performed in the epithelial compartments. MYC protein and mRNA quantification was reported previously for these cases (51). Total original magnification, ×200. *n* = 12 patients.

Prior work has shown that the cyclical treatment of men with mCRPC treated with supraphysiological levels of testosterone (supraphysiological androgen [SPA]), referred to as bipolar androgen therapy (BAT), produces tumor regression and clinical benefit in ~30%–40% of patients (51, 67–71). Many of the samples from men with mCRPC shown in Figure 2 were from the pretreatment time point of a recent trial, referred to as COMBAT-CRPC (51) (clinicaltrials.gov; NCT03554317), where patients received a pretreatment biopsy of a metastatic site as well as a biopsy after 3 cycles of SPA (cycle 4, day 1 [C4D1]). Interestingly, most patients in the trial who responded to SPA also showed a marked reduction in MYC protein expression, by quantitative IHC, as well as a marked reduction MYC mRNA by RNA-Seq. This was accompanied by reductions in Ki67 protein as well as a number of proliferation-associated mRNAs (51, 72). We performed the mtDNAcn CISH-IHC assay on all available samples from patients with mCRPC that had matched posttreatment biopsies from COMBAT-CRPC. Figure 6 shows that there was a decrease in mtDNAcn that correlated with decreases in MYC protein in most cases. These findings provide evidence that, in late-stage mCRPC, changes in mtDNAcn after drug treatment correlate with changes in MYC levels, further indicating that MYC may be a key regulator of mtDNAcn in prostatic adenocarcinomas.

### mtDNA replication as a potential downstream target of MYC in prostate cancer.

**Figure 7:**
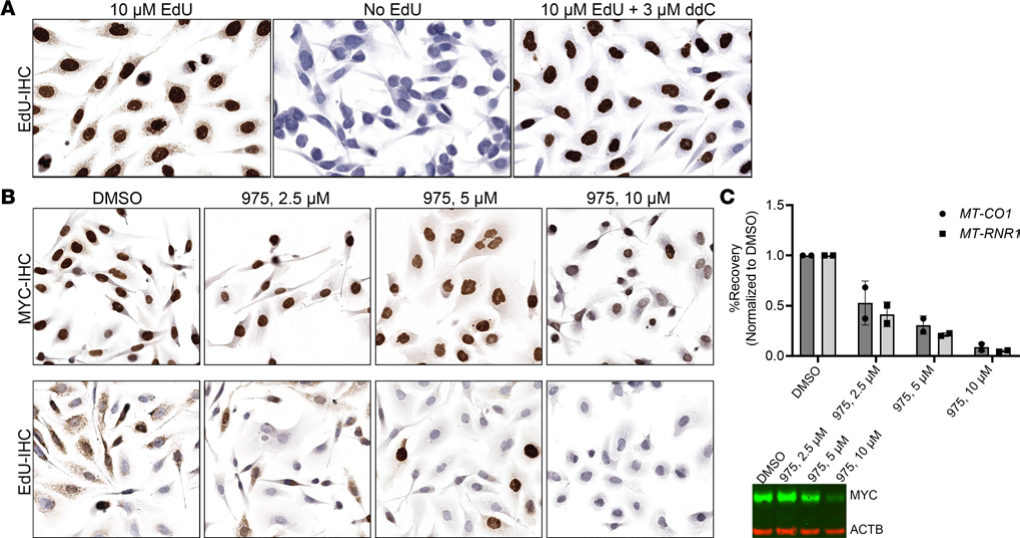
MYC activity controls mitochondrial DNA synthesis. (**A**) Representative images showing that the anti-BrdU antibody staining is only present after PC3 cells are pretreated with EdU overnight, demonstrating its specificity for EdU. Cells grown on chamber slides were metabolically labeled with EdU or no EdU and were then fixed and stained by IHC. Note the complete absence of staining in the middle panel in cells without EdU treatment and the marked reduction in cytoplasmic signals after treatment with the mtDNA replication inhibitor, ddC (right panel). (**B** and **C**) When MYC activity and/or levels were decreased using MYCi975, decreased EdU (after 4 hour incubation) incorporation into cytoplasmic or mitochondrial foci was observed by IHC (**B**) and separately by EdU immunoprecipitation followed by qPCR in PC3 cells (**C**). *n* = 2 independent biological replicates in IP. Representative Western blot results confirmed the decrease in MYC after drug treatment (**C**) in the samples subjected to IP. Total original magnification for IHC images, ×400 (**A**), ×200 (**B**).

To address whether MYC can drive mtDNA replication in prostate cancer cells (PC3), we inhibited MYC activity with a potentially novel small molecule inhibitor MYCi975 (73, 74), followed by metabolic labeling with EdU for incorporation into newly synthesized DNA. Using an anti–5-bromodeoxyuridine (anti–5-BrdU) antibody that also binds to EdU (75) (Figure 7A), we visualized the EdU signals by IHC. In untreated cells, we observed abundant punctate dot-like structures in the cytoplasm, consistent with mitochondrial localization. As a control for antibody specificity, these IHC signals were absent in cells not treated with EdU. In support of the hypothesis that these signals reflect mtDNA replication, the EdU-IHC signals were greatly reduced in cells treated with the mtDNA polymerase (POLG) inhibitor ddC. Many cells also showed strong nuclear signals for EdU, indicating nuclear S phase labeling (Figure 7A). After inhibition of MYC by MYCi975, there were reduced cytoplasmic signals, consistent with a decrease in mtDNA replication. There was also a decrease in nuclear DNA (nDNA) signals after MYC inhibition, which is expected since loss of MYC will also block nDNA synthesis in prostate cancer cells (Figure 7B) (76). As a second approach, we performed qPCR for mtDNA and nDNA after immunoprecipitation of EdU containing DNA. Using this assay, we also observed decreased EdU incorporation into mitochondrial genes when MYC levels were reduced (Figure 7C; see complete unedited blots in the supplemental material). Taken together, these results support the hypothesis that MYC activity controls mtDNA synthesis in prostate cancer cells.

### mtDNA replication factors in prostate cancer.

**Figure 8:**
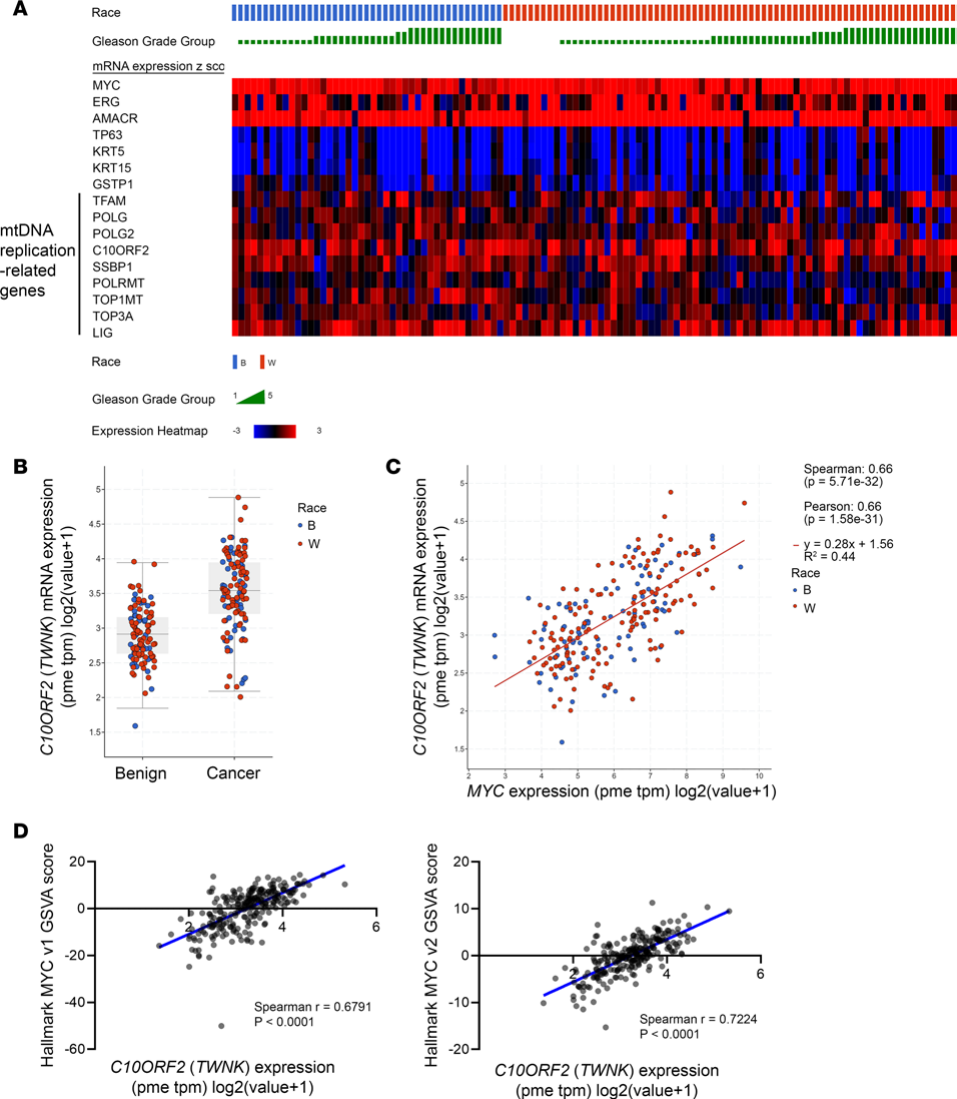
Genes encoding proteins required for mtDNA replication are upregulated in prostate cancer from human laser-capture microdissected samples as determined by RNA-Seq. (**A**) Heatmap showing upregulation of transcripts in genes encoding mtDNA replication factors. Top line shows the Gleason Grade group, and the second line shows the patient race. *ERG* and *AMACR* serve as controls for known upregulated genes in prostate cancer, and *TP63*, *KRT5*, *KRT15*, and *GSTP1* serve as controls for known downregulated (basal cell enriched) genes in prostate cancer. Genes directly involved in mtDNA replication are *TFAM*, *POLG* (mtDNA polymerase), *POLG2*, *C10ORF2* (*TWNK*), *SSBP1*, *LIG3*, *PRIMPOL*, and *TOP3A*. (**B**) *TWNK/C10ORF2* mRNA was increased in cancer compared with benign. Boxes show medians with interquartile ranges; whiskers indicate 1.5 × interquartile range. Wilcoxon test was used. *P* < 0.0001). (**C**) Correlation of *TWNK/C10ORF2* mRNA with *MYC* mRNA. (**D**) Correlation of *TWNK/C10ORF2* with Hallmark MYC pathway scores.

Genes required for mtDNA replication, which are all encoded by nDNA, include *POLG* (the main DNA polymerase), *POLG2* (a processivity subunit), *TWNK* (the helicase referred to as Twinkle), *SSBP1* (the mtDNA specific single stranded binding protein), *TFAM* (encoding mitochondrial transcription factor A required for replication and transcription), *LIG3* (the DNA ligase), *PRIMPOL* (the DNA primase-polymerase), *TOP1MT* (encoding the mitochondrial topoisomerase), and *TOP3A* (a mitochondrial isoform encoding topoisomerase required to deconcatenate mtDNA circles). Germline mutations in the genes that encode these proteins lead to mtDNA depletion syndromes (77). A number of these genes have been previously shown to be MYC targets in other cell types (53, 78). To examine the potential regulation of these genes in prostate cancer by MYC, we interrogated mRNA levels of these genes after siRNA knockdown of MYC in 3 prostate cancer cell lines (76). Most of the mtDNA replication genes were reduced after MYC knockdown (Supplemental Figure 6A). Publicly available ChIP-Seq showed that MYC binding occurs within the promoter regions of multiple mtDNA replisome genes in a prostate cancer cell line and that this binding is reduced after MYC inhibitor treatment; RNA-Seq data show that their transcripts were reduced correspondingly (74) (Supplemental Figure 6B). Furthermore, we examined the RNA-Seq data mentioned above from the same samples that were subjected to LCM and whole-genome sequencing. Figure 8 shows increased mRNA in primary prostate cancer lesions compared with matched benign lesions for a number of genes encoding these mtDNA replication factors, especially Twinkle (*TWNK/C10ORF2*). Furthermore, a number of these factors, including Twinkle, correlate with MYC expression in these primary tumors (Figure 8C). When we examined RNA-Seq data from the COMBAT CRPC trial discussed above (51), a similar correlation with *MYC* and *TWNK* was present (Supplemental Figure 6C). This correlation between *MYC* and *TWNK* mRNA levels was also found in other cancer types in publicly available TCGA data sets, including those from human breast carcinoma, pancreatic adenocarcinoma, and colorectal carcinoma (Supplemental Figure 7). These findings suggest that a number of the necessary components of mtDNA replication machinery are positively regulated by MYC, are bound in their regulatory regions by MYC protein, are overexpressed in human prostatic cancer, and correlate with MYC levels in primary tumors and in mCRPC. To further explore whether TWNK overexpression relates to MYC-driven transcriptional activity more generally, we compared TWNK mRNA levels with the Hallmark MYC Pathways V1 and V2 using gene set variation analysis (GSVA) and found a strong positive correlation between these (Figure 8D).

We also sought to determine whether expression of nuclear genes encoding proteins that localize and function in mitochondria were increased in human prostate cancers. For this, we performed GSEA using the Mitocarta 3.0 mitochondrial pathways and found that many of these were enriched in prostate cancer in our U01 samples (samples part of an NIH-U01–funded study), a number of which were considered statistically significant (e.g., mitochondrial_central_dogma.Translation, mitochondrial_central_dogma.mtRNA_metabolism, mitochondrial_central_dogma.Protein_homeostatsis) (Supplemental Figure 8).

### Increased mtDNAcn was also seen in other cancer precursor lesions.

**Figure 9:**
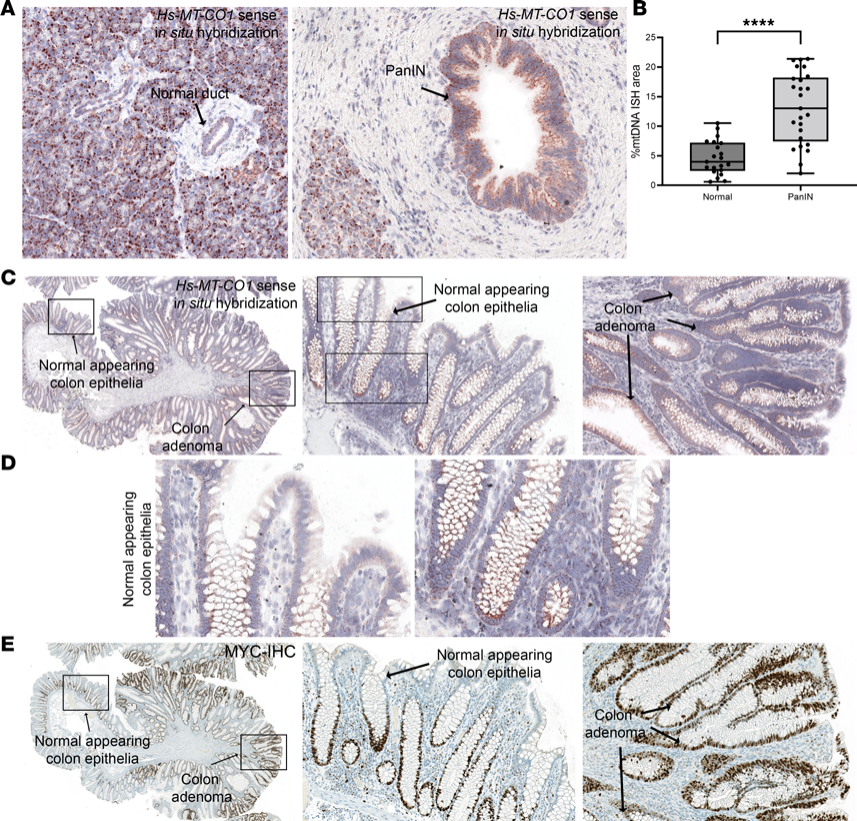
Increased mtDNAcn is commonly found in other cancer precursor lesions. (**A**) Representative ISH results of a normal pancreatic duct versus a PanIN. Note that the relatively high mtDNAcn seen does not reflect high levels of proliferation in the normal-appearing pancreatic acinar cells but likely reflects the high energetic needs of these cells. (**B**) Quantification of mtDNAcn in normal ducts and PanINs using image analysis. Each symbol represents a region of the tissue type specified. Mann-Whitney *U* test was used. ****P < 0.0001. (**C**–**E**) Representative images of mtDNA ISH and MYC IHC in a colon sample containing an adenomatous polyp. Boxed areas in the left-most images in **C** and **E** are shown as higher magnification in the middle and right panels. Boxed areas in the middle panel of **C** are shown in **D** to illustrate the differential mtDNA levels along the surface (left) versus bases (right) of colonic crypts. Total original magnification, ×200 (**A**), ×20 (**C** and **D**), ×40 (middle and right panels in **C** and **E**), and ×400 (**D**). *n* = 11 and 8 for patients for PanIN and colon adenoma, respectively.

To determine the applicability of our in situ approach in other cancer precursor lesions, we investigated mtDNAcn in precancerous lesions from the pancreas and large intestine. We applied the same CISH-IHC workflow on human pancreas tissues with pancreatic intraepithelial neoplasia (PanIN) and benign pancreatic ducts; we also applied this workflow on human colon tissues with tubular adenoma and normal colon mucosa. By visual inspection, PanIN lesions showed higher mtDNA ISH signals compared with their normal duct counterparts (Figure 9A). By image analysis, we observed an approximately 3-fold higher percentage of mtDNA area in PanIN (%mtDNA area) compared with the normal ducts (Figure 9B). In the colonic samples, as recently reported (48), when present, the normal-appearing colonic epithelium showed a marked gradation in mtDNAcn with cells located in the stem/proliferative compartments of crypts showing higher mtDNAcn compared with more differentiated cells toward the surface. In 7 of 8 adenomas, by visual inspection, the mtDNA signals were more similar to, or apparently higher than, the levels observed in the normal-appearing stem cell compartment (Figure 9, C and D). These strong mtDNA signals occurred even toward the surface of the adenomatous epithelium — including in MYC protein expression — in a manner reminiscent of topographical infidelity of proliferation markers and other proteins in colorectal adenomas (Figure 9E) (79, 80).

## Discussion

To overcome the limitations inherent in most mtDNAcn measurements in tumor/normal pairs, we developed a multiplex CISH and IHC approach to quantify mtDNAcn in specific cell populations. This assay uncovered consistent increases in mtDNAcn in high-grade PIN. In addition, we found increases and marked intratumoral heterogeneity in mtDNAcn in most primary prostatic adenocarcinomas and further increases in advanced mCRPC. The increased mtDNAcn in primary prostate cancer was independently verified using whole-genome sequencing (WGS) of DNA isolated after LCM as well as by using qPCR. Since 3 separate complementary approaches produced highly similar results, our findings should resolve previous contradictory publications regarding differences in mtDNAcn in prostatic cancers as compared with matched benign normal-appearing prostate epithelium. The increased mtDNAcn in prostate cancer cells associated with increases in mtRNAs and mitochondrial electron transport chain activity, observed by in situ enzymatic activity, suggests increased mitochondrial function. Mechanistically, increased mtDNAcn in mouse prostatic luminal epithelial cells in vivo is driven by forced overexpression of MYC. Additionally, reductions in mtDNAcn correlate with reductions in MYC that are observed in mCRPC clinical samples after treatment with supraphysiological testosterone (51). Since MYC is known to be consistently overexpressed in human PIN (compared with normal prostate luminal cells) and most primary and metastatic castration-resistant prostatic adenocarcinomas (51, 52, 56, 63), these findings implicate MYC overexpression as causing mtDNAcn increases in these lesions. We also report that a key mechanism by which MYC drives increased mtDNAcn is through increased mtDNA replication, potentially via positive regulation of mtDNA replication factors, including the helicase, TWINKLE. Finally, our in situ approach also identified increases in mtDNAcn in neoplastic precursor lesions in the colon (adenomas) and pancreas (PanINs), demonstrating the broad applicability of this method.

### Comparisons with other prostate cancer studies.

Our findings are consistent with the findings of Mizumachi et al. (43). Our in situ method and their single-cell LCM approach both measure mtDNAcn with single-cell resolution. Our findings also generally agree with a more recent study using whole-genome sequencing data from TCGA samples that showed a trend for increased mtDNAcn in prostate cancer versus benign matched tissues (45). Although Reznik et al. reported that there was no correlation between mtDNAcn and mtRNA expression in prostate cancer using sequencing methods (81), we observed that, at least for the 3 mtRNAs, their spatial patterns and expression levels highly correlate with those of mtDNAcn. The present findings differ with those of Schöpf et al. (18), who found no overall increase in mtDNAcn in cancer versus normal in the prostate, although they did report an increase in mtDNAcn in higher-grade tumors as compared with the lower-grade ones. By contrast, we found consistently increased mtDNAcn across all grade groups. Nevertheless, in our study, the Gleason Grades of all of the cases with the highest levels of mtDNA in primary tumors were greater than Gleason Grade group 1, and we found a further increase in metastatic castration-resistant lesions (Figures 2 and 3). Schöpf et al. (18) used tissue-punch biopsies to isolate tumor and benign tissues, which provides a lower level of tumor purity than our laser-capture–based method used for our WGS approach, and this could help explain the differences. Our findings differed from those of Koochekpour et al. (44), since we found no difference in Black versus White men in tumor mtDNAcn, while their study did.

### Basal versus luminal cells.

In the present study, we found that basal cells have approximately 5-fold higher %mtDNA area as compared with normal luminal cells. This indicates a higher mtDNA density in basal cells compared with luminal cells. In comparing mtDNA cellular levels in HGPIN and invasive adenocarcinomas with normal levels, there was a clear increase overall, even when including normal basal cells in the normal regions, suggesting a higher mtDNA density per unit cellular area (Figure 2). By visual inspection, there was a clear increase in mtDNA in situ signals in luminal cells in PIN and cancer compared with normal luminal cells. Although some prostatic adenocarcinomas appear to possess gene expression signatures, or modules, that resemble basal cells to a degree (82–84), most evidence suggests that the cell of origin for prostate carcinoma is a luminal cell (56, 85–87). When compared specifically with benign normal-appearing luminal cells, there was a more striking increase in HGPIN luminal cells (Supplemental Figure 9).

### MYC may drive mtDNA replication.

Prior studies have shown that MYC can regulate mitochondrial biogenesis and mtDNA synthesis (53, 78). Wang et al., reported that during *Drosophila* oogenesis, *myc* regulated mtDNA replication and COX enzyme activity, while a hypomorphic *myc* allele led to reduced levels of both (78). They further suggested that such changes may occur through myc’s role as a master transcription factor, since a majority of the genes encoding proteins of the electron transport chain, mtDNA replication, and transcription were downregulated in flies with the hypomorphic *myc* alleles. Our results mirrored theirs; we observed similar trends when examining microarray data after knockdown of MYC in human prostate cancer cell lines, and we observed RNA-Seq results from a clinical trial where responding patients showed reduced MYC was accompanied reduced mRNA levels in genes such as *TWNK* (Supplemental Figure 6). Furthermore, a number of mtDNA replication–related genes are upregulated in human prostate cancer, and *TWNK* levels correlate with *MYC* mRNA (Figure 8 and Supplemental Figure 6C). Taken together, these findings suggest an essential role of MYC in mtDNA replication and mitochondrial function in both *Drosophila* normal ovarian tissues and prostate cancer development and progression. Further study is required to assess whether increased mtDNAcn (or de novo mtDNA synthesis) is required for tumor development and progression in MYC-driven cancers.

### Why mtDNAcn is increased.

We hypothesize that increased mtDNAcn is necessary in the process of carcinogenesis (40, 41). For instance, it is possible that higher mtDNAcn drives mitochondrial biogenesis to increase mitochondrial activity needed for the increase in macromolecular synthesis required for cell growth and replication. That there may be increased mitochondrial biogenesis in prostate cancer is further supported by electron microscopy studies that showed apparently elevated mitochondrial numbers in human prostate cancer samples (88–90) as well as in PIN (referred to as intraductal dysplasia; ref. 91). Since our mCRPC samples generally showed increased mtDNAcn compared with primary prostate cancer, we hypothesize that the cells with high mtDNAcn have a growth advantage and, thus, may be selected during disease progression. In support of this, we found reduced mtDNAcn in matched samples from patients before and after SPA that correlated with clinical response, Ki67 levels, and MYC levels. Studies over several decades have indicated that, compared with normal prostate, prostate cancer cells show increased mitochondrial functions that are important for cell proliferation, and our overall results are concordant with these observations (92–96).

An alternative hypothesis is that, since mtDNA mutations are common in prostate cancer (10, 15, 18, 81), some of which may be deleterious to mitochondrial functions, increased mtDNAcn may reflect compensatory upregulation of mtDNA levels to help maintain these compromised mitochondrial functions. There is a potential precedent for this in prostate cancer, as seen by Schöpf et al., in which they found higher mtDNAcn in cases showing potentially deleterious mtDNA mutations (18). In that study, short-term cultures of fresh prostate cancer tissue showed an altered mitochondrial oxidative phosphorylation capacity, including a decrease in N-pathway (NADH through Complex I, III, IV) capacity and an increase in S-pathway (succinate from Complex II-IV) capacity, which was accentuated in cases with higher levels of heteroplasmy of potentially deleterious mtDNA mutations in proteins encoding Complex I components (18). Furthermore, mtDNA mutations, or nuclear-encoded genes that can alter the TCA cycle flux, can result in increases in oncometabolites that can drive epigenetic changes, such as DNA CpG methylation, that can provide a selective growth advantage to the neoplastic cells (25), despite having somewhat compromised overall mitochondrial function. Thus, in this case, there could also be compensatory increases in mtDNAcn to maintain mitochondrial function.

One additional limitation of our study is that, although we were able to quantify mtDNAcn within specific cell compartments, we did not examine this at the single-cell level. Future studies that employ cell membrane markers for combined in situ and IHC analysis, as well as potential future methods that can perform single-cell mtDNA sequencing/copy number measurements, could help address the potential mtDNAcn heterogeneity at the single-cell level.

### Translational relevance and future directions.

The discovery of functional mitochondrial reprogramming geared for biomass generation and proliferation in many cancers (1) has nominated pharmacological blockade of mitochondrial oxidative phosphorylation, mtDNA replication, and mitochondrial translation (28, 97) as promising and novel anti-cancer therapeutic avenues. Based upon the present findings, we hypothesize that developing novel drugs to inhibit required mtDNA replication machinery components, including the helicase Twinkle (encoded by *TWNK*), SSBP1, and mitochondrial topoisomerases, may represent new therapeutic approaches for cancer treatment and/or prevention. This concept is analogous to the recent credentialing of POLMRT, the mtRNA polymerase, as a novel cancer drug target (98). Related to this, it has been hypothesized that those tumors with high levels of activity of canonical mitochondrial functions, such as TCA cycle and oxidative phosphorylation, may harbor dependencies that could be most susceptible to mitochondrial inhibition therapies (27, 37, 99). Since mtDNAcn regulation is associated with controlling mitochondrial biogenesis and function, accurate measurements of mtDNAcn in tumors and their precursors using our in situ approach may help determine which cancers are most appropriate for mitochondrial therapies. Also, the fact that we observed elevated mtDNAcn in precursor lesions from 3 different tumor types, and that others have seen this in head and neck cancers (49), suggests that blockade of mitochondrial activities may be an important new approach for precision prevention and cancer interception strategies. Future methods that can measure mtDNA using single-cell–based sequencing methods may also have an important role here (100), although they would lose critical spatial information that is a key feature in our approach. The method developed in this study is straightforward and should be readily portable to automated in situ assays combined with IHC, when appropriate, that could be implemented routinely in research laboratories and clinical pathology laboratories if additional future studies support clinical utility.

## Methods

### Mice

Hi-MYC mice (64) on an FVB/N background (*n* = 3, age = 2–6 months) were conventionally housed, and animal tissues were harvested and fixed in 10% neutral buffered formalin for 48 hours.

### Chromogenic mtDNA ISH combined with IHC

ISH for mtDNA for fresh frozen and formalin-fixed paraffin-embedded (FFPE) tissues was performed based on a previous method for human prostates and colons (48). For human pancreas FFPE tissues, the pretreatment was 15-minute steaming in RNAscope Target Retrieval reagent followed by 10-minute incubation in protease plus at 40°C; the rest is the same as human prostate and colon tissues and others in ref. 48.

The mtDNA ISH signals were developed using 3-amino-9-ethylcarbazole (AEC; Vector Laboratories, SK-4200), counterstained in hematoxylin, and stained blue in Shandon bluing buffer (Thermo Fisher Scientific, 6769001). The slides were mounted, coverslipped, scanned, and decoverslipped. The AEC was destained in an alcohol gradient. The slides were then rehydrated in an alcohol gradient and incubated using a HRP blocker (Advanced Cell Diagnostics) for 15 minutes at 40°C before being subjected to sequential IHC. This scanning, destaining, and blocking process was repeated between all staining cycles.

After ISH, the slides were incubated with an anti-CK8 antibody (1:1500, Abcam, ab53280) for 45 minutes and PowerVision Poly-HRP anti–rabbit IgG (Leica, PV6114) for 30 minutes, and they were visualized by incubation in AEC for 30 minutes, followed by counterstaining and bluing. For some slides, an additional CK903 IHC staining cycle was performed after CK8 detection. CK903 (1:50, Enzo, ENZ-C34903) was used as the primary antibody, PowerVision Poly-HRP anti–mouse IgG (Leica, PV6119) as the secondary antibody, and AEC as the chromagen. All primary antibodies were diluted in Antibody Dilution Buffer (Ventana, adb250) unless specified.

The mtDNA ISH on mouse prostate tissues using *Mm-mt-Co1* sense probe was performed as described previously (48). *Hs-MYC* mRNA ISH was performed similarly with probes against human *MYC* (Advanced Cell Diagnostics, 311761).

### Multiplex fluorescence ISH and immunofluorescence

Multiplex fluorescence ISH for mitochondrial RNA expression was performed using HiPlex12 detection kit (Advanced Cell Diagnostics, 324105) on fresh frozen tissues. Briefly, fresh frozen prostate tissues were hybridized with RNAscope HiPlex probes against *Hs-MT-CO1* sense (mtDNAcn), *Hs-MT-CYB*, *Hs-MT-ATP6*, and *Hs-MT-RNR1* in 2 rounds. Whole slides were scanned, and fluorophores were cleaved between rounds. After HiPlex, the slides were then incubated in anti-CK8 and anti-CK903 antibodies with the same concentration as above. Secondary antibodies were the same, except they were used at 1:125 dilution. Lastly, the slides were developed with Opal fluorescent dye at 1:50 dilution (Akoya Biosciences) in different channels. Catalogs for HiPlex probes are as follows: *Hs-MT-CO1* sense (478051), *Hs-MT-CYB* (582771), *Hs-MT-ATP6* (532961), and *Hs-MT-RNR1* (425961), all from Advanced Cell Diagnostics.

### IHC

All single-plex DAB-based chromogenic IHC on tissue samples (FFPE or fresh frozen) was performed on a Ventana Discovery ULTRA system (Roche). Fresh frozen tissues were air dried at room temperature for 15 minutes, fixed overnight in formalin, and rinsed off with dH_2_O prior to staining. IHC labeling for c-Myc and ERG was performed with primary antibodies against c-MYC (1:600, Abcam, ab32072) and ERG (EPR3864) (Roche, 6478450001), respectively. IHC for EdU and MYC on cells grown on chamber slides was performed as described (48). A primary antibody against BrdU (Abcam, ab136650) was validated to be specific for EdU as well, and used at 1:1,000. The primary antibody against c-MYC was used at 1:250 dilution (Abcam, ab32072). The secondary antibodies were PowerVision Poly-HRP anti-rabbit or anti-mouse (Leica, PV6114 and PV6119, respectively) and developed with DAB.

### Image analysis

Whole slides were scanned at ×40 (0.25 μm per pixel) on a Ventana DP200 slide scanner (Roche) for chromogenic slides, or a TissueFAXS slide scanner for fluorescent slides (TissueGnostics). All the image processing and analyses were performed using HALO version 3.0 (Indica labs). Single-stained whole slide images in the chromogenic multiplex ISH and IHC assays were fused using the Serial Staining Image Fusion protocol provided by HALO (54). Briefly, after scanning, each set of images were registered. Images were color deconvolved in a case-by-case manner based on red, green, blue (RGB) optical density values for each marker in AEC and for hematoxylin. During this process, the registration transforms generated in the previous step were used to align all slides into the same coordinate system. The pseudocolor single-stained whole slide images were then fused with the same image resolution as the original images and then subjected to downstream analyses. Fluorescence scanning images were exported using TissueFAXS viewer software (TissueGnostics) in single-channel images and fused similarly in HALO.

A random forest algorithm in the Tissue Classifier Add-on of HALO was used to distinguish epithelium from stroma and empty tissue compartments. The calculated area of each compartment type was based on the CK8 and hematoxylin status for each slide. CK8 was chosen, since it is positive for all normal prostatic epithelial cells, PIN, and primary and metastatic prostate cancer. In needle biopsies from metastatic sites in the liver, in which hepatocytes are CK8^+^, the metastatic carcinoma regions were manually outlined. For samples that were also stained with CK903, the classifier was additionally trained to recognize and calculate the area of prostate basal epithelium versus luminal epithelium. Classifiers were trained for each slide individually. All classifiers had resolution set at 1.04 μm/px and minimum object size at 20 μm^2^. For radical prostatectomy samples, regions of normal prostatic epithelium, HGPIN, and prostate cancer were annotated before analysis. To quantify mtDNA ISH signals, the Area Quantification FL algorithm (v.2.1.2) and above-mentioned classifiers were used in combination, and %mtDNA area in each classified compartment (e.g., prostate basal cells, prostate luminal cells, or total prostate epithelium [basal plus luminal cells]) were measured and reported. Since we observed that many of the single-punctate hybridization signals (dots) appeared to coalesce into larger punctae, instead of counting individual puncta, we quantified the overall area of mtDNA signals as related to the tissue compartments. This %mtDNA area is strongly correlated with mtDNAcn, as defined by the ratio of mtDNA levels to nDNA levels (48). The quantification algorithm of each image was individually optimized using the real-time tuning function based on both the deconvoluted image and the original chromogenic image.

### Cell lines and cell culture

PC3, LNCaP, and CWR22Rv1 prostate cancer cells (ATCC) were cultured in RPMI 1640 medium supplemented with 10% (v/v) FBS at 37°C and 5% CO_2_. Cells were maintained on cell culture flasks (Sarstedt) or CC2 chamber slides (Thermo Fisher Scientific, 154739). MYCi975 was originally reported by Han et al. (73) and was obtained from AxonMedChem (Axon 3229).

### Western blotting

Whole cell extracts from cell lines were obtained using RIPA buffer (Thermo Fisher Scientific) containing a protease inhibitor cocktail (Roche) and benzonase (Thermo Fisher Scientific). Protein concentrations were quantified using a BCA gold assay (Thermo Fisher Scientific). Equal amounts of total protein were prepared in 4× NuPAGE LDS Sample Buffer (NP0007, Invitrogen) and 5% BME and were boiled at 95°C for 5 minutes. The protein samples were resolved on 4%–12% NuPAGE Bis-Tris gel with protein molecular weight standards (PageRuler Prestained Protein Ladder, 10–180 kDa, Thermo Fisher Scientific). The gels were transferred onto PVDF membranes using the Trans-Blot Turbo Transfer System (Bio-Rad). Primary antibodies used in this study included: rabbit anti–c-Myc antibody [Y69] (ab32072, Abcam, 1:1,000) and mouse anti–β-actin antibody (3700, Cell Signaling, 1:5,000). Fluorescent secondary antibodies used in this study were IRDye 680RD donkey anti–mouse IgG secondary antibody (LI-COR, 926-68072, 1:5,000) and IRDye 800CW donkey anti–rabbit IgG secondary antibody (LI-COR, 926-32213, 1:5,000).

### EdU-immunoprecipitated DNA and qPCR

To measure newly synthesized mtDNA, we developed a method for EdU-immunoprecipitated DNA and qPCR based on a similar approach by Jiang et al. (101). Briefly, cells grown on flasks were treated with MYCi975 at the indicated concentrations for 48–96 hours. Before harvesting, the cells were incubated in cell culture media containing 10 μM EdU for 4 hours or overnight. Cells were divided into 2 portions, 1 for total DNA extraction and the other for total protein extraction. Total DNA was extracted using DNeasy blood and tissue kit (Qiagen). Immunoprecipitation of EdU-containing DNA was performed using the iDeal ChIP-Seq kit for Transcription Factors (Diagenode) with the following modifications. For each immunoprecipitation (IP) reaction, 30 μL of washed Protein A–coated magnetic beads was combined with 70 μL IP reaction mix containing 6 μL of BSA, 20 μL 5x iC1b buffer, and 0.5 μg antibody in ChIP grade water and incubated for 2 hours at 4°C under rotation. Equal amounts of total DNA (5–15 μg) were added to the reaction mix and incubated overnight at 4°C under rotation. The beads were washed and the DNA was eluted and purified by following the manufacturer’s protocol. One percent of the INPUT, which corresponds to whole DNA that went through the full IP procedure without any immunoselection, was subjected to the same elution and purification process. DNA (1 μL) from the purified IP and INPUT samples was used for qPCR. TaqMan Universal PCR Master Mix II (no UNG) and TaqMan probes (mitochondrial: *MT-RNR1*, *MT-CO1*; nuclear: *BGLT3*) were used following the manufacturer’s protocol (Thermo Fisher Scientific). EdU-containing newly synthesized DNA was calculated and reported by comparing the relative amount of IP DNA to the INPUT DNA for specific mitochondrial and nuclear genes (percentage of recovery). The anti-BrdU/EdU antibody (Abcam, ab136650) was used, and mouse (G3A1) mAb IgG1 (Cell Signaling Technology, 5415) was used as the negative isotype–matched control for the IP reactions.

### mtDNAcn and RNA-Seq in laser-capture microdissected prostate tissues

Fresh frozen tissues were placed in OCT and sections were cut at 7 μm thickness and mounted onto membrane slides (Leica, 11600288). LCM of regions of interest was performed using Leica LMD 7000 Microscope in which study pathologists were careful to exclude areas with overt inflammatory infiltrates, carcinoma, and high-grade PIN when isolating normal-appearing regions. Tissue digestion and DNA/RNA extraction was performed using Allprep DNA/RNA Kits following the manufacturer’s recommendations (Qiagen, 802804). Sequencing was performed using Illumina HiSeqX and NovaSeq6000 with Paired End 150 bp × 150 bp read configuration. Trim galore v0.6.3 was used to trim the reads. Bwa v0.7.7 (mem) was used to align to the hg19 and hg38 human genome builds. Piccard tools v1.119 and GATK v3.6.0 were used to create a recalibrated bam file. Bedtools v2.27.1 (genomecov) was used to determine the nuclear coverage (i.e., all chromosomes except mtDNA) and the mitochondrial coverage. mtDNAcn was computed using the following formula: mitochondrial coverage/nuclear coverage. RNA-Seq and gene expression measures on mtDNA replication related genes were performed as previously described (102). The analysis of the WGS and RNA-Seq data here is limited to the reported mtDNAcn assessment. Full details and reporting of the WGS data will be reported as part of another study.

### Statistics

Statistical analysis and visualization was performed using Prism GraphPad 9.0 and R version 4.0.2. For box-and-whiskers plots, unless specified in the figure legend, boxes present medians with interquartile ranges, and whiskers present minimum to maximum values; all points are shown. Statistical analysis between 2 groups was performed using Mann-Whitney *U* test for nonpaired samples and Wilcoxon test for paired samples. For more than 2 sample comparisons, Kruskal-Wallis 1-way analysis was used.

### Study approval

#### Human studies.

Human prostate tissues (frozen, or formalin fixed and paraffin embedded [FFPE]) were obtained from radical prostatectomies performed at The Johns Hopkins Hospital. Their use was approved by the Johns Hopkins University School of Medicine IRB (NA_00087094). Supplemental Table 1 summarizes the clinical features of the cases used for ISH study. Supplemental Table 2 summarizes the clinical features of the patients participating in the whole-genome sequencing and RNA-Seq study. These samples were part of an NIH-U01–funded study that will be reported upon in detail separately and are referred to as the U01 samples. Organ donor prostates were obtained from the National Disease Research Interchange (NDRI). For each organ donor specimen, the entire prostate was submitted for histology and was histologically confirmed to be free of invasive adenocarcinoma as well as other significant pathology.

#### Animal studies.

All animal study protocols were approved by the IACUC at Johns Hopkins University School of Medicine.

### Data availability

Publicly available ChIP-Seq and RNA-Seq data on cells treated with MYCi975 were obtained from NCBI (Gene Expression Omnibus [GEO] accession nos. GSM5399497 GSM5399571, GSM5399627, and GSM5399631) (74) and visualized using the Integrated Genomics Viewer (IGV). RNA-Seq data on *MYC* and *TWNK* in different cancer types from TCGA PanCancer Atlas was accessed using cBioportal (103, 104). The RNA-Seq data from laser-capture microdissected prostate tissues described above have been deposited in NCBI’s GEO and are accessible through GEO accession no. GSE246067.

## Author contributions

JC, SY, CVD, and AMDM conceptualized and designed the project. JC, QZ, JLH, LT, BO, TJ, AMV, TCL, RW, MCM, SRD, KJP, RHH, ESA, AG, SY, and AMDM acquired, analyzed, and interpreted data. JC and AMDM wrote the original draft of the manuscript. All authors were responsible for reviewing and editing the manuscript.

## Supplementary Material

Supplemental data

Supporting data values

## Version 1 11/16/2023

In-Press Preview

## Version 2 12/22/2023

Electronic publication
