# Supplementary material for: MYC-driven increases in mitochondrial DNA copy number occur early and persist throughout prostatic cancer progression
Source: JCI Insight. 2023 Dec 22;8(24):e169868. doi: 10.1172/jci.insight.169868 (PMC10807718; doi:10.1172/jci.insight.169868)
Supplement: Supplemental data [file jciinsight-8-169868-s127.pdf]

Supplemental Methods

Detailed mtDNA copy number and RNAseq in laser capture microdissected prostate tissues

Fresh frozen tissues were placed in OCT and sections were cut at 7 µm thickness and mounted onto membrane slides (Leica, 11600288). The slides were fixed in ice-cold 70% EtOH for 5 min, hydrated in RNAse free water, counterstained in hematoxylin for 20 seconds, washed twice in RNAse-free water, and air dried. The slides were stored at 4°C for a short time before microdissection. LCM of regions of interest was performed using Leica LMD 7000 Microscope in which study pathologists were careful to exclude areas with overt inflammatory infiltrates, carcinoma, and high grade PIN when isolating normal appearing regions. Tissue digestion and DNA/RNA extraction was performed using Allprep DNA/RNA Kits following the manufacturer's recommendations (Qiagen, 802804). For DNA library preparation, samples were sheared by sonication on the Covaris S2 System; libraries were constructed according to the protocol provided in the Illumina Nano TruSeq Library prep guide. Assessment of the yield and size distribution of the amplified library was performed on the Agilent 2100 Bioanalyzer using the High Sensitivity Chip. Libraries were then pooled at equal molar concentrations for sequencing. Sequencing was performed using Illumina HiSeqX and NovaSeq6000 with Paired End 150 bp x 150 bp read configuration. For sequencing data processing, trimgalore v0.6.3 was used to trim the reads. Bwa v0.7.7 (mem) was used to align to the hg19 and hg38 human genome builds. Piccard-tools v1.119 & GATK v3.6.0 were used to create a recalibrated bam file. Bedtools v2.27.1 (genomecov) was used to determine the nuclear coverage (i.e., all chromosomes except mtDNA) and the mitochondrial coverage. mtDNA copy number was computed using the following formula: mitochondrial coverage/nuclear coverage. RNAseq and gene expression measures on mtDNA replication related genes were performed as previously described (102). The analysis of the WGS and RNA-seq data here is limited to just the reported mtDNA copy number assessment and the

gene expression measures for known mtDNA replication genes. Full details and reporting of the WGS and RNA-seq data will be reported as part of another study.

Differential gene expression analysis and gene set enrichment analysis

Differential gene expression analysis and pathway analysis on RNAseq data from laser capture microdissected prostate samples were performed using DESeq2 (103) and GSVA with z-score method (104). Gene set enrichment analysis on microarray data from cell lines with MYC knockdown (76) was performed using the fgsea R package (105). The mtDNA replication related gene set was obtained from the Mitocarta 3.0 database (106).

Quantitative PCR

Quantitative PCR to measure mtDNAcn from DNA extracted from LCM prostate samples was conducted as described previously (48). Probes used in this study were Hs-MT-CO1 (Thermo Fisher, Hs4331182) as the mitochondrial gene and BGLT3 (Thermo Fisher, Hs01629437) as the nuclear gene reference.

COX and SDH enzyme histochemistry

COX and SDH enzyme histochemistry was performed on fresh frozen human prostate tissues. Briefly, fresh prostate tissues were collected, frozen, cryosectioned into 5 μm (SDH) or 10 μm (COX) sections. The frozen slides were air dried at room temperature for 1 hour. COX reaction mix containing 25 μL of 4 mM Cytochrome c, 67 μL of 10 mg/mL 3,3′-diaminobenzidine tetrahydrochloride (DAB) in 1 mL 1X PBS was freshly prepared, followed by addition of 2 μg bovine catalase and vortexing, and added to slides for 40 min at 37 °C in a moist chamber. SDH reaction mix was prepared using 1.5 mM NBT, 130 mM sodium succinate, 0.2 mM PMS, and 1.0 mM sodium azide in 1X PBS. The mixture was applied to tissue slides for 40 min at 37 °C in a

moist chamber. The slides were washed in PBS for 4 x 10 min, dehydrated in an ethanol gradient and xylene, mounted and coverslipped in Cytoseal 60.

Supplemental Figures and Supplemental Figure Legends

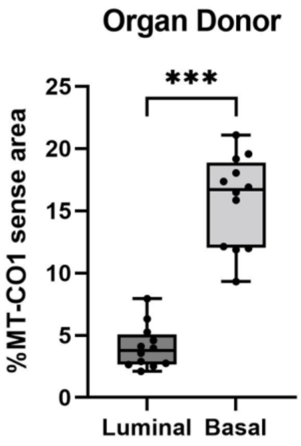

Supplemental Figure 1. Image analysis results showing higher mtDNAcn in normal basal cells compared to normal luminal cells in organ donor prostates. The center line in the box shows the median %mtDNA area in each group. N = 4 regions from 3 organ donors for each cell type. Wilcoxon test, ***P = 0.0005.

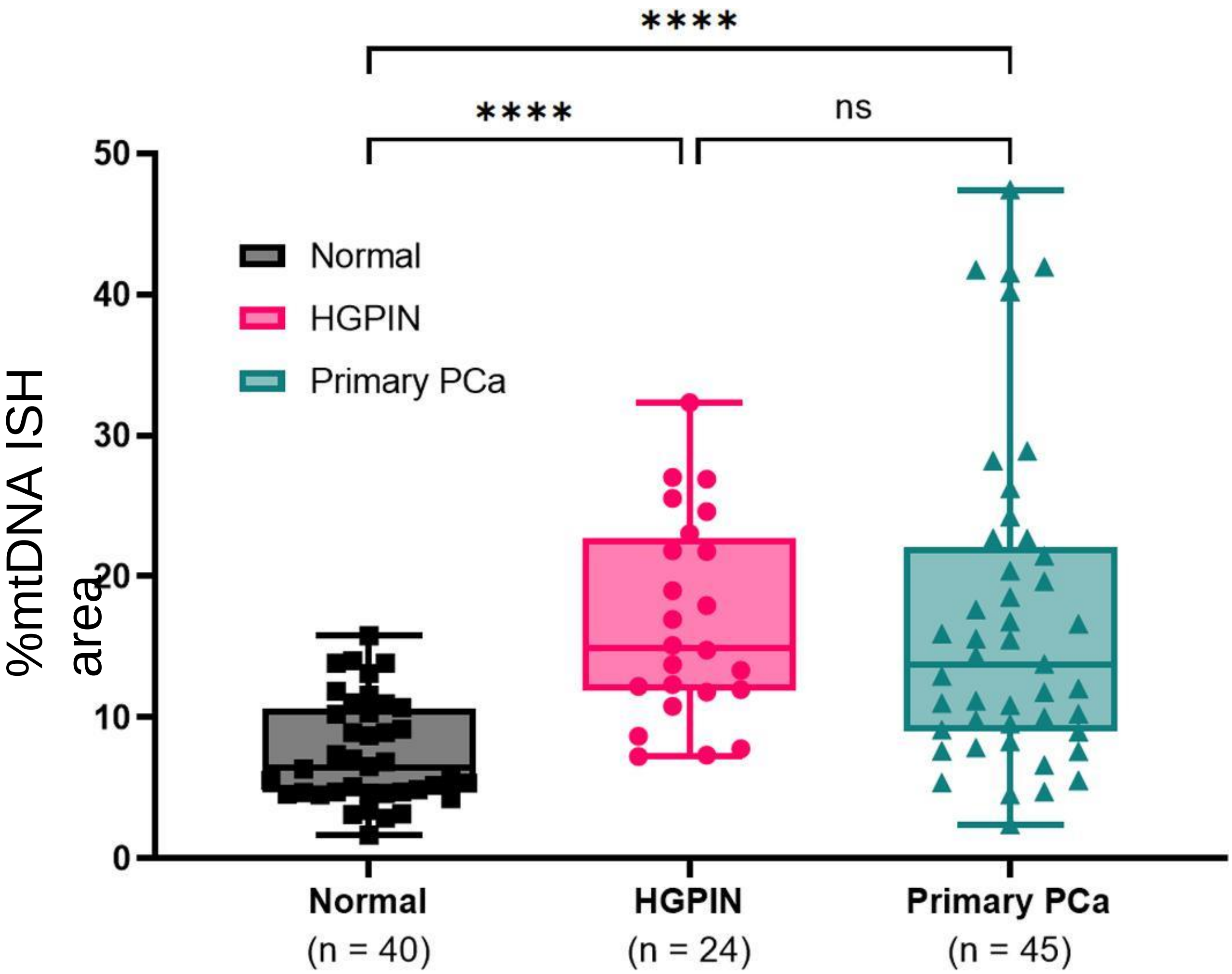

Supplemental Figure 2. **Heterogeneity of mtDNAcn in human prostate.** Quantitative image analysis results from individual regions of normal, HGPIN and primary prostate carcinoma. Each symbol represents an individual region of interest examined. N = 40, 24, 45 for normal, HGPIN, and Primary PCa lesions. Kruskal-Wallis test followed by Dunn's test for nonparametric pairwise multiple comparisons, ****P < 0.0001.

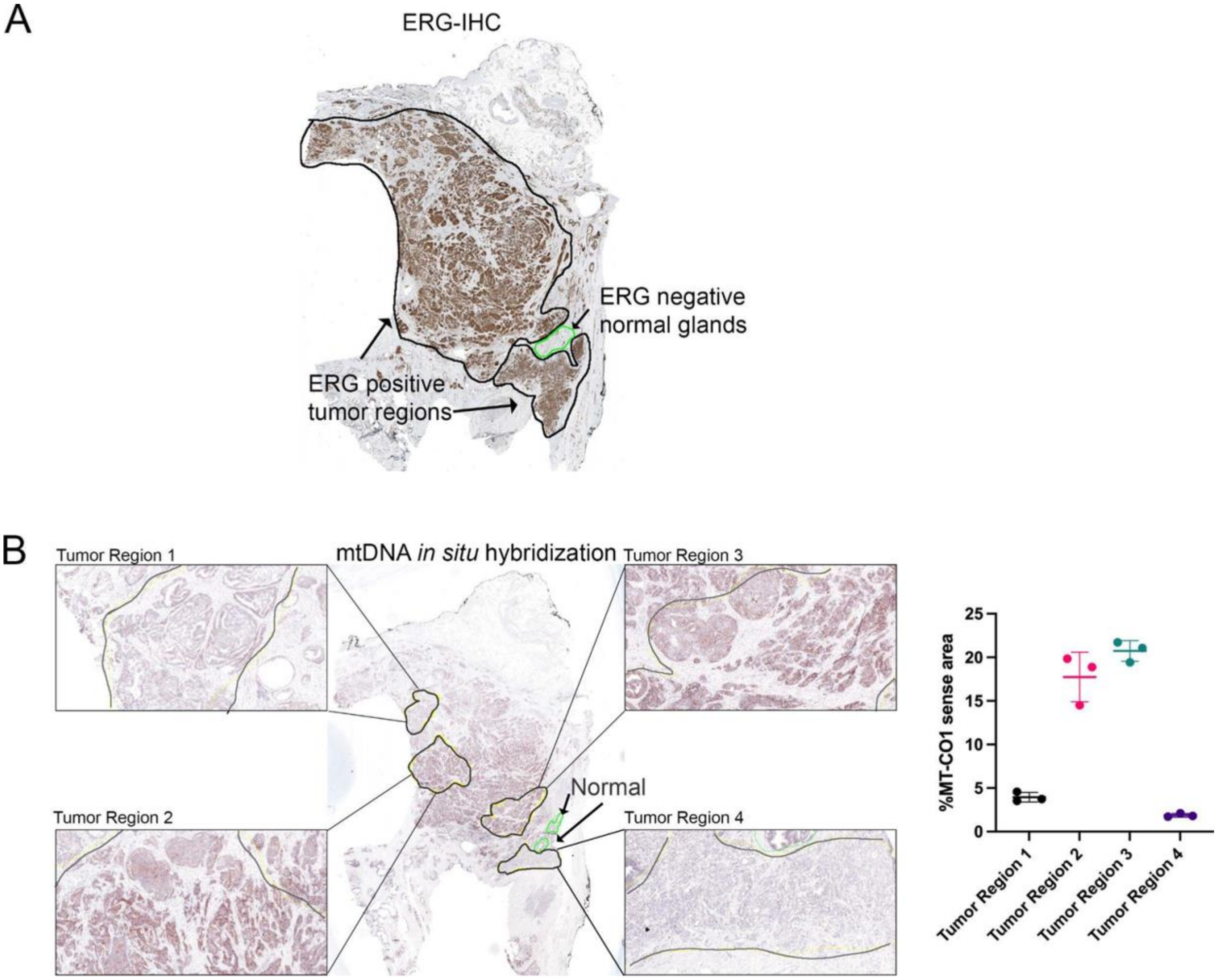

Supplemental Figure 3. Intratumoral heterogeneity in a single prostatic tumor nodule. (**A**) Low power view of a prostatectomy specimen with tumor nodule that is homogeneously positive for ERG protein, indicative of an *ERG* gene fusion. Original magnification, x10. (**B**) Marked heterogeneity of mtDNA *in situ* hybridization signals from different subregions of this tumor using an adjacent slide to that shown in **A** and corresponding quantitative image analysis results supporting statistically significant heterogeneity among different tumor regions. Kruskal-Wallis test, P = 0.0003. Original magnification, x10 and x70 (boxed regions).

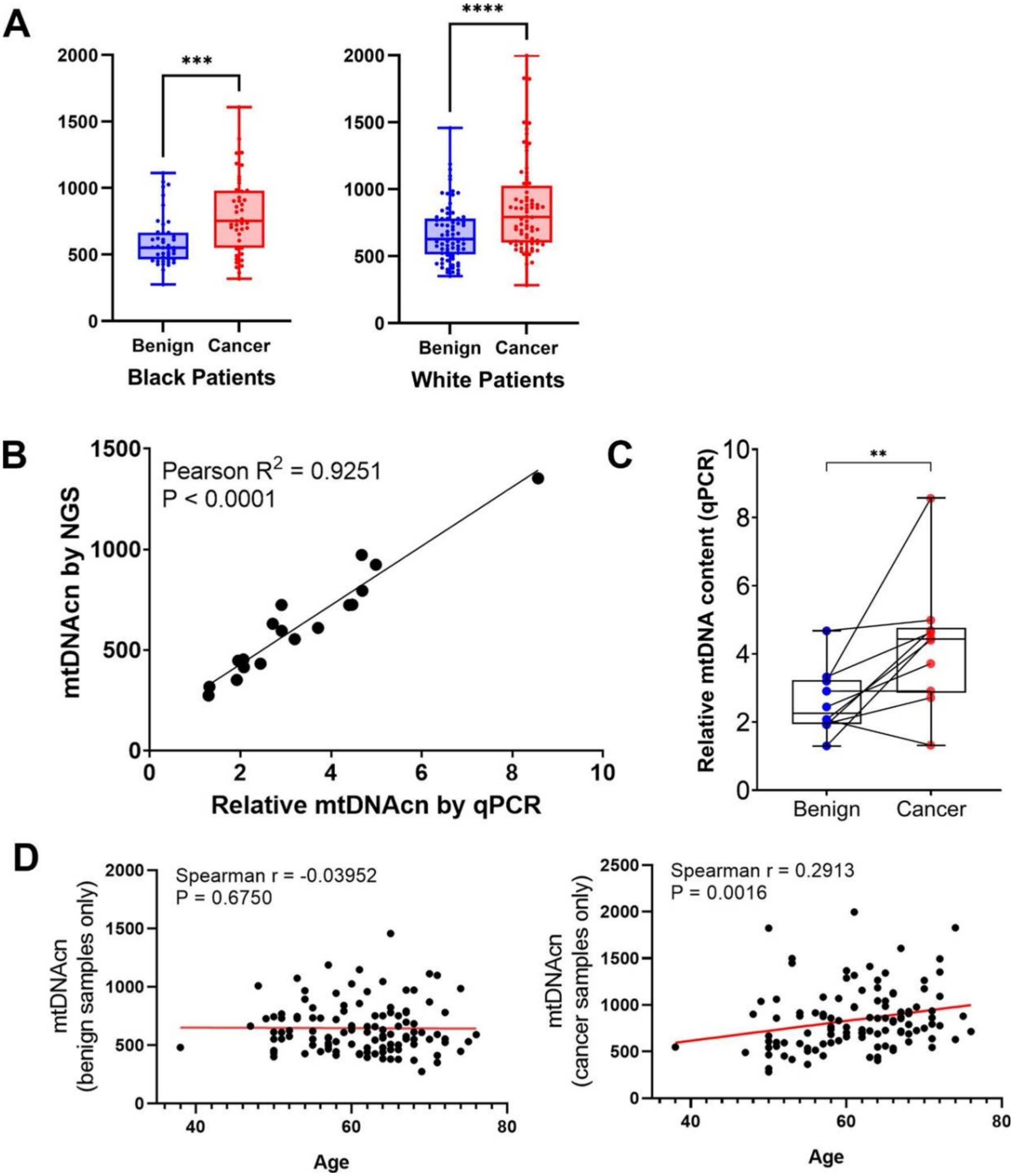

Supplemental Figure 4. **Increased mtDNAcn occurs in prostatic adenocarcinoma tissues from both Black and White men.** (**A**) Similar increases in mtDNAcn were found in tumors compared to matched benign prostate samples from Black and White men after WGS. N = 43 and 72 for Black and White Patients, respectively. Wilcoxon test, ***P = 0.0003 for Black patients, and **** P < 0.0001 for White patients. (**B**) Scatter plot shows a strong correlation of mtDNA levels when comparing the results from WGS to qPCR using the same prostate samples from LCM. (**C**) Relative mtDNAcn by qPCR shows a similar pattern of increased levels in tumor tissue to the

results found by *in situ* hybridization (Fig. 2) and WGS (Fig. 3) (n = 10 patients). Wilcoxon test, **P = 0.0098. (**D**) Correlation between age and mtDNAcn was observed in prostate cancer samples.

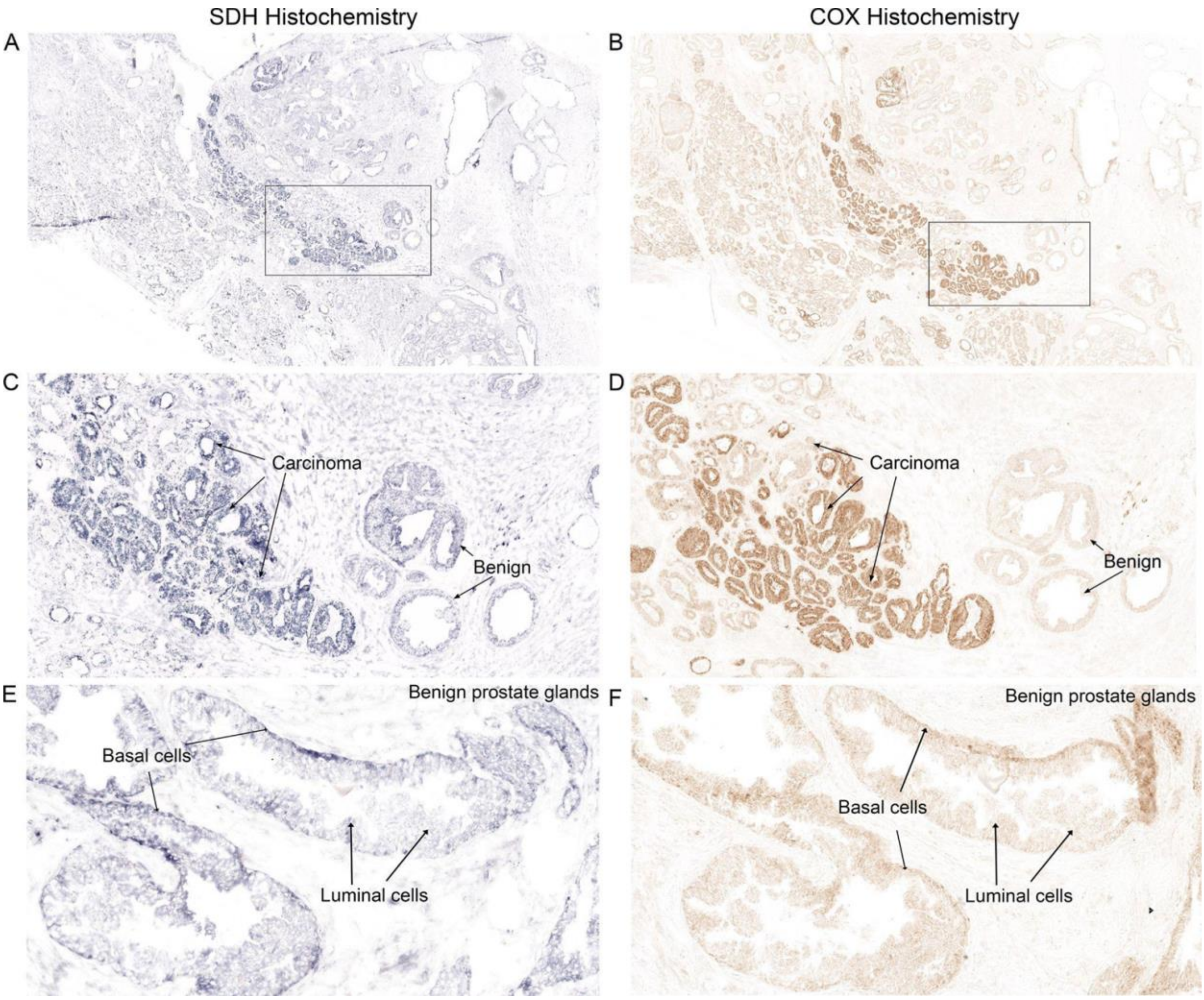

Supplemental Figure 5. Increased COX and SDH enzyme activity is observed in prostate cancer compared to normal adjacent glands. (**A** and **C**) SDH histochemistry and (**B** and **D**) COX histochemistry both reveal increased and heterogenous signals in the cancer regions, similar to the results of mtDNA *in situ* hybridization. **E** and **F** are representative images showing higher SDH and COX enzymatic activities in normal basal cells compared to the normal luminal cells in a different area from the same samples as in **A**-**D**. Original magnification, x20 (**A** and **B**), x70 (**C** and **D**) and x200 (**E** and **F**). N = 11 patients.

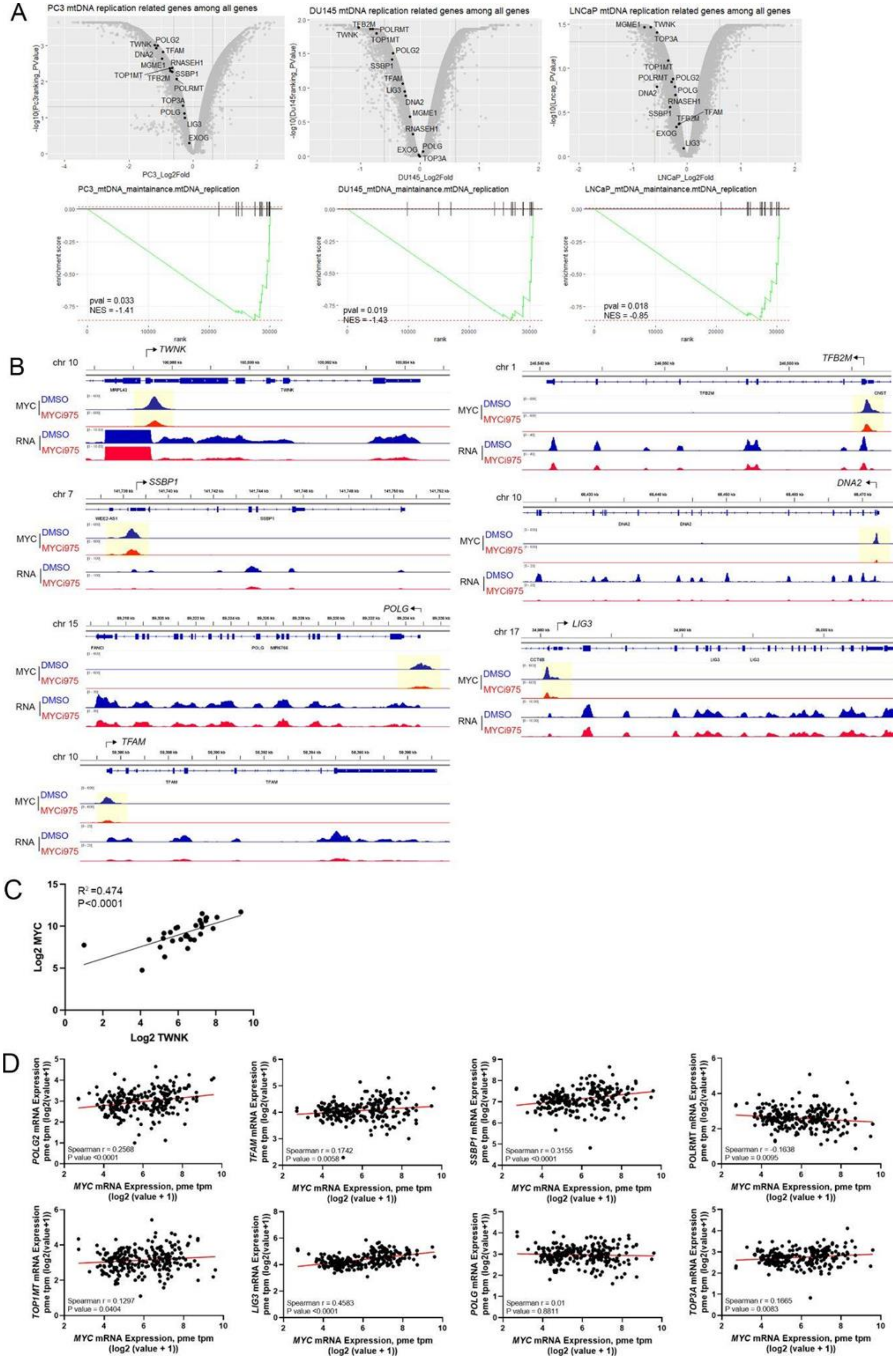
A
PC3 mtDNA replication related genes among all genes
DU145 mtDNA replication related genes among all genes
LNCaP mtDNA replication related genes among all genes
PC3_Log2Fold
DU145_Log2Fold
LNCaP_Log2Fold
PC3_mtDNA_maintainance.mtDNA_replication
DU145_mtDNA_maintainance.mtDNA_replication
LNCaP_mtDNA_maintainance.mtDNA_replication
enrichment score
rank
pval = 0.033
NES = -1.41
pval = 0.019
NES = -1.43
pval = 0.018
NES = -0.85
TWNK
POLG2
TFAM
DNA2
RNASEH1
MGME1
SSBP1
TOP1MT
TFB2M
POLRMT
TOP3A
POLG
LIG3
EXOG
B
chr 10
chr 7
chr 15
chr 1
chr 17
MYC
RNA
DMSO
MYCi975
C
R² =0.474
P<0.0001
Log2 MYC
Log2 TWNK
D
POLG2 mRNA Expression pme tpm (log2(value+1))
Spearman r = 0.2568
P value <0.0001
TFAM mRNA Expression pme tpm (log2(value+1))
Spearman r = 0.1742
P value = 0.0058
SSBP1 mRNA Expression pme tpm (log2(value+1))
Spearman r = 0.3155
P value <0.0001
POLRMT mRNA Expression pme tpm (log2(value+1))
Spearman r = -0.1638
P value = 0.0095
TOP1MT mRNA Expression pme tpm (log2(value+1))
Spearman r = 0.1297
P value = 0.0404
LIG3 mRNA Expression pme tpm (log2(value+1))
Spearman r = 0.4583
P value <0.0001
POLG mRNA Expression pme tpm (log2(value+1))
Spearman r = 0.01
P value = 0.8811
TOP3A mRNA Expression pme tpm (log2(value+1))
Spearman r = 0.1665
P value = 0.0083
MYC mRNA Expression, pme tpm (log2 (value + 1))

Supplemental Figure 6. MYC regulates a number of genes encoding factors involved in mtDNA replication. (**A**) Microarray analysis in cell lines after siRNA-based MYC knockdown (59) reveals down regulation of mtDNA replication-related genes from 3 different human prostate cancer cell lines. (**B**) Publicly available ChIP-seq and RNA-seq data using 22Rv1 prostate cancer cells shows MYC protein binds to the promoter region of genes encoding proteins for mtDNA replication, and MYC occupancy and expression levels were decreased after the cells were treated with a MYC inhibitor. (**C**) Correlation between *MYC* and *TWNK* (encoding TWINKLE) in mCRPC cases in the COMBAT-CRPC study. Each point represents the Log2 mRNA TPM from RNAseq from laser captured frozen tissue biopsy samples. (**D**) Positive correlations between *MYC* and many other mtDNA replication related genes were observed by RNAseq using the U01 study samples.

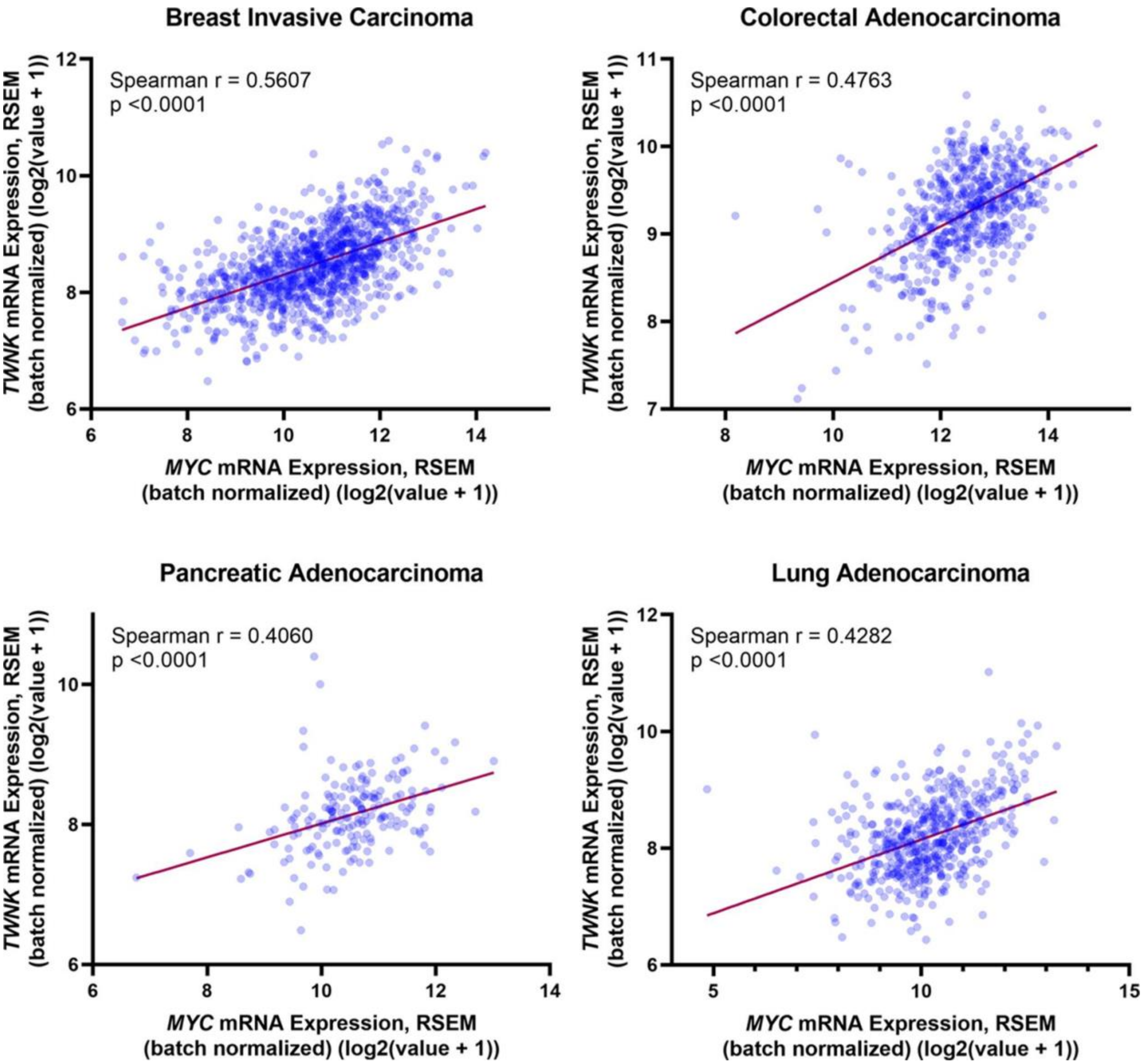

Supplemental Figure 7. Correlation between *TWNK* and *MYC* RNA expression in various cancer types. *TWNK* expression level is correlated with *MYC* expression in multiple cancers. Lines indicate the best fit linear trend between these two transcripts.

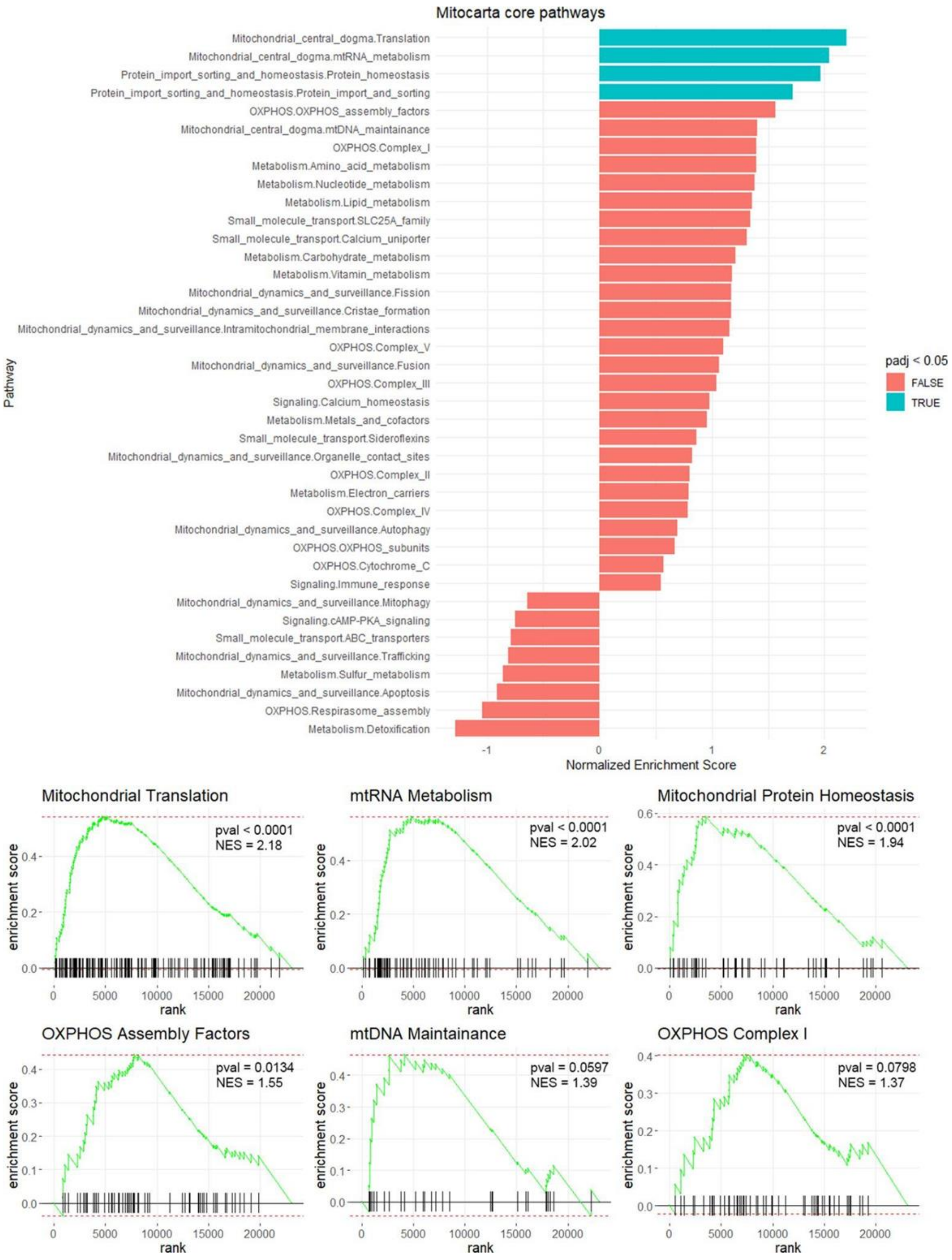
Mitocarta core pathways
Mitochondrial_central_dogma.Translation
Mitochondrial_central_dogma.mtRNA_metabolism
Protein_import_sorting_and_homeostasis.Protein_homeostasis
Protein_import_sorting_and_homeostasis.Protein_import_and_sorting
OXPHOS.OXPHOS_assembly_factors
Mitochondrial_central_dogma.mtDNA_maintainance
OXPHOS.Complex_I
Metabolism.Amino_acid_metabolism
Metabolism.Nucleotide_metabolism
Metabolism.Lipid_metabolism
Small_molecule_transport.SLC25A_family
Small_molecule_transport.Calcium_uniporter
Metabolism.Carbohydrate_metabolism
Metabolism.Vitamin_metabolism
Mitochondrial_dynamics_and_surveillance.Fission
Mitochondrial_dynamics_and_surveillance.Cristae_formation
Mitochondrial_dynamics_and_surveillance.Intramitochondrial_membrane_interactions
OXPHOS.Complex_V
Mitochondrial_dynamics_and_surveillance.Fusion
OXPHOS.Complex_III
Signaling.Calcium_homeostasis
Metabolism.Metals_and_cofactors
Small_molecule_transport.Sideroflexins
Mitochondrial_dynamics_and_surveillance.Organelle_contact_sites
OXPHOS.Complex_II
Metabolism.Electron_carriers
OXPHOS.Complex_IV
Mitochondrial_dynamics_and_surveillance.Autophagy
OXPHOS.OXPHOS_subunits
OXPHOS.Cytochrome_C
Signaling.Immune_response
Mitochondrial_dynamics_and_surveillance.Mitophagy
Signaling.cAMP-PKA_signaling
Small_molecule_transport.ABC_transporters
Mitochondrial_dynamics_and_surveillance.Trafficking
Metabolism.Sulfur_metabolism
Mitochondrial_dynamics_and_surveillance.Apoptosis
OXPHOS.Respirasome_assembly
Metabolism.Detoxification
Pathway
padj < 0.05
FALSE
TRUE
-1
0
1
2
Normalized Enrichment Score
Mitochondrial Translation
pval < 0.0001
NES = 2.18
mtRNA Metabolism
pval < 0.0001
NES = 2.02
Mitochondrial Protein Homeostasis
pval < 0.0001
NES = 1.94
OXPHOS Assembly Factors
pval = 0.0134
NES = 1.55
mtDNA Maintainance
pval = 0.0597
NES = 1.39
OXPHOS Complex I
pval = 0.0798
NES = 1.37
enrichment score
rank

Supplemental Figure 8. Many mitochondrial related pathways were upregulated in prostate cancer samples. GSEA analysis was performed on RNAseq data from U01 samples after differential gene expression analysis using DESeq2 and FGSEA.

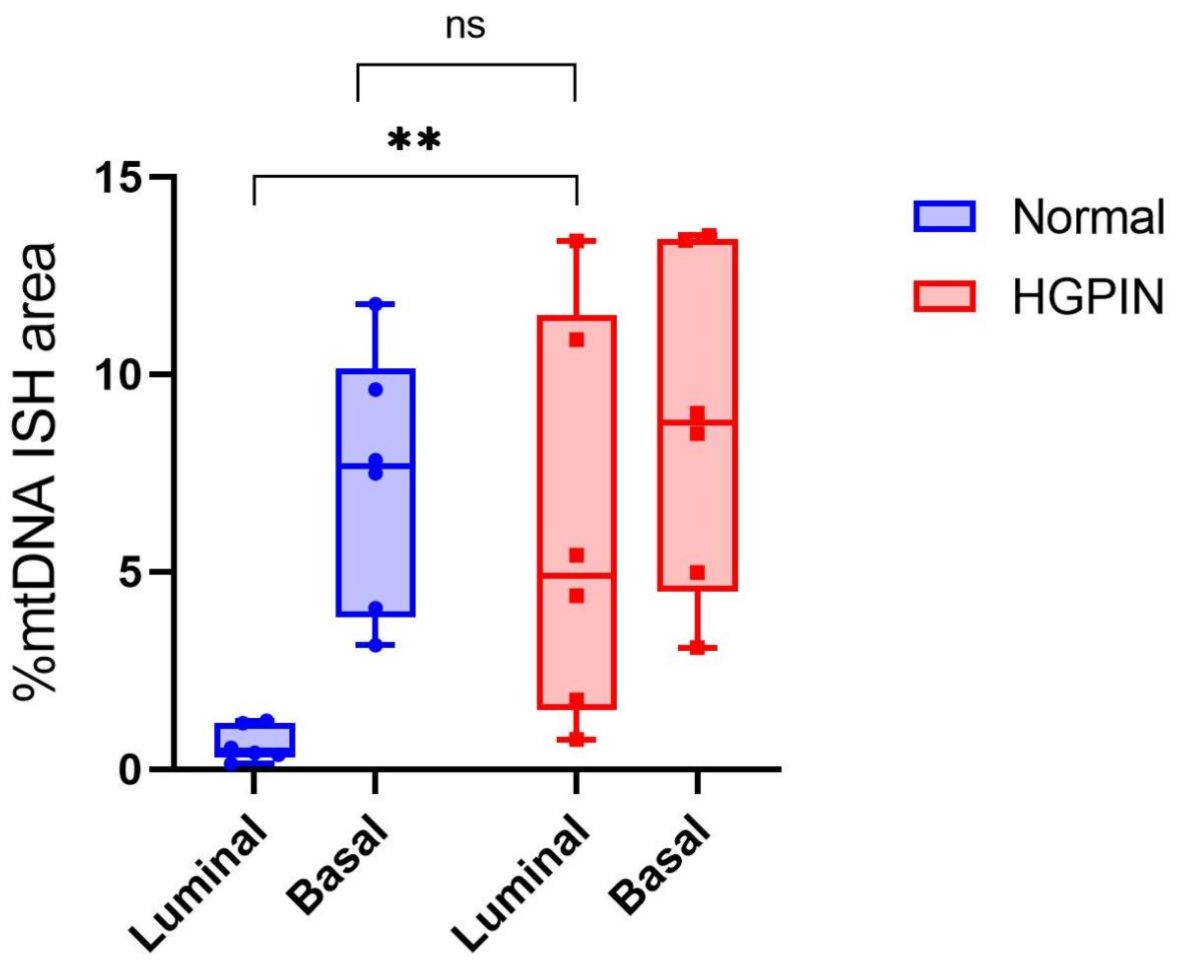

Supplemental Figure 9. HGPIN luminal cells exhibit higher mtDNA *in situ* hybridization signals than normal luminal cells in the human prostate. Tissues were subjected to multiplex CISH-IHC as in Fig. 1. Quantitative image analysis results from individual regions of normal and HGPIN glands in which luminal and basal cells were assessed separately. Non-parametric Mann-Whitney tests were done on luminal cells between HGPIN and normal as well as basal cells in normal vs. luminal cells in HGPIN. n = 2 regions per tissue type from 3 patients. **P = 0.0087.

Supplemental Table 1. Pathology of Cases used in Chromogenic *in situ* hybridization for mtDNAcn.

Pt Num	Age Range	Race*	Margins	Grade Group	Gleason Primary	Gleason Secondary	Gleason Sum	Gleason Tertiary	P Stage**
1	60-69	W	Negative	2	3	4	7		T2N0MX
2	60-69	W	Negative	2	3	4	7		T3AN0MX
3	70-79	B	Positive	5	4	5	9		T3BN0MX
4	60-69	W	Positive	2	3	4	7		T2XNXMX
5	60-69	B	Negative	3	4	3	7	5	T3AN0MX
6	50-59	W	Positive	4	4	4	8		T3BN0MX
7	50-59	W	Positive	3	4	3	7	5	T3BN0MX
8	<50	W	Negative	5	4	5	9		T3BN1MX
9	50-59	W	Negative	5	4	5	9		T3BN1MX
10	70-79	W	Positive	3	3	4	7		T2N0MX
11	50-59	W	Negative	3	3	4	7		T2N0MX
12	60-69	W	Negative	3	3	4	7		T3AN0MX
13	60-69	W	Negative	2	3	4	7	4	T2NXMX
14	60-69	W	Positive	2	3	4	7		T2XN0MX
15	50-59	W	Negative	5	4	5	9		T3AN0MX
16	60-69	B	Positive	2	3	4	7		T3ANXMX
17	60-69	W	Positive	3	4	3	7		T3AN0MX

* Race is self identified. B = Black patients, W = White patients.
** P Stage is the pathological stage at radical prostatectomy using the American Joint Committee on Cancer Staging 2007.

Supplemental Table 2. Demographic and pathological features of 115 patients with the laser captured microdissected prostate samples for whole genome sequencing and RNAseq.

Age	*B	W	Total
**<50**	3	1	4
**50-59**	13	28	41
**60-69**	23	31	54
**70-79**	4	12	16
**Total**	43	72	115

	****Pathological Stage**				
**Grade group**	**T2**	**T3A**	**T3BN0**	**Any T, N1**	**Total**
**1**	9	1	0	0	10
**2**	20	14	0	2	36
**3**	9	11	7	2	29
**4**	3	3	1	0	7
**5**	5	11	10	7	33
**Total**	46	40	18	11	115

* Race is self identified. B = Black patients, W = White patients.
** P Stage is the pathological stage at radical prostatectomy using the American Joint Committee on Cancer Staging 2007.

Supplemental Table 3. mtDNAcn in matched benign and cancer samples from 115 patients with the laser captured microdissected prostate samples for whole genome sequencing.

Patient number	Benign mtDNAcn	Cancer mtDNAcn	Race	Age Group	Gleason Score	Gleason Grade Group
Pt 1	516.377	902.0808	B	50-59	6	1
Pt 2	613.22185	698.40325	B	60-69	9	5
Pt 3	621.54855	504.63825	B	50-59	7	3
Pt 4	752.2564	1414.2173	W	60-69	9	5
Pt 5	396.097595	701.2713	W	60-69	9	5
Pt 6	414.728335	738.701	W	50-59	7	3
Pt 7	370.11901	790.84445	W	60-69	7	2
Pt 8	502.68945	537.19375	W	60-69	9	5
Pt 9	453.346605	317.88646	B	50-59	7	3
Pt 10	552.85165	1823.3479	W	50-59	8	4
Pt 11	425.993355	722.5421	B	60-69	7	3
Pt 12	508.70365	659.6069	W	60-69	7	2
Pt 13	558.84125	876.54185	B	50-59	7	3
Pt 14	444.053355	594.78635	W	50-59	7	2
Pt 15	500.62055	362.83962	B	50-59	7	2
Pt 16	793.2603	720.1457	W	60-69	7	2
Pt 17	732.51765	915.6952	W	50-59	7	3
Pt 18	554.6214	598.3444	W	50-59	7	3
Pt 19	274.50324	725.21725	B	60-69	8	4
Pt 20	552.7797	535.9851	W	50-59	7	2
Pt 21	721.39745	595.7057	W	50-59	6	1

Pt 22	780.2323	1494.1411	W	70-79	9	5
Pt 23	576.4304	868.183	W	60-69	7	3
Pt 24	506.86145	986.49295	B	60-69	9	5
Pt 25	446.648715	515.6884	W	50-59	6	1
Pt 26	1457.95515	1341.76985	W	60-69	7	3
Pt 27	592.03305	937.3867	W	70-79	9	5
Pt 28	575.1221	594.9942	W	50-59	7	3
Pt 29	379.882475	513.5554	W	60-69	9	5
Pt 30	743.9985	542.9445	W	50-59	9	5
Pt 31	778.5899	728.3215	W	60-69	7	2
Pt 32	592.6602	543.98185	B	70-79	7	3
Pt 33	668.74725	1129.38215	W	60-69	7	2
Pt 34	427.750025	586.8138	W	50-59	7	3
Pt 35	522.66425	1092.3098	W	70-79	7	3
Pt 36	413.08681	639.3081	W	70-79	7	3
Pt 37	384.98502	404.23511	B	60-69	8	4
Pt 38	630.37495	761.56145	W	60-69	7	2
Pt 39	432.22977	610.399	W	50-59	7	3
Pt 40	351.614165	794.5014	W	70-79	7	3
Pt 41	591.60105	716.4147	W	70-79	8	4
Pt 42	824.5358	1042.1809	W	60-69	7	2
Pt 43	530.7829	880.07975	W	70-79	9	5
Pt 44	554.1587	1352.99715	W	70-79	8	4

Pt 45	447.60045	630.78655	W	70-79	9	5
Pt 46	858.26385	1316.7659	W	60-69	9	5
Pt 47	625.4827	452.109545	W	50-59	7	3
Pt 48	438.825665	652.57265	B	60-69	7	3
Pt 49	441.360395	1183.548	B	60-69	9	5
Pt 50	752.0692	1044.91855	W	60-69	10	5
Pt 51	659.14605	865.63385	W	60-69	7	3
Pt 52	613.00755	282.81053	W	50-59	7	3
Pt 53	579.0205	857.35905	W	60-69	7	2
Pt 54	381.587965	558.96975	W	60-69	9	5
Pt 55	820.2923	536.4771	W	50-59	7	2
Pt 56	608.7008	617.25735	W	50-59	7	2
Pt 57	400.1153	668.32325	W	50-59	6	1
Pt 58	986.4316	1829.1012	W	70-79	9	5
Pt 59	562.77525	440.78738	W	60-69	7	2
Pt 60	972.50795	923.96355	W	60-69	7	3
Pt 61	608.3548	977.56915	W	60-69	6	1
Pt 62	698.25255	708.32505	W	60-69	6	1
Pt 63	781.1871	854.77275	W	60-69	7	2
Pt 64	420.20485	980.74915	B	60-69	9	5
Pt 65	481.57116	549.0752	W	<50	7	2
Pt 66	523.9648	777.884	W	70-79	6	1
Pt 67	662.1022	1120.7465	W	60-69	7	2

Pt 68	468.15402	888.8268	W	50-59	6	1
Pt 69	734.76075	698.47725	W	50-59	7	2
Pt 70	894.53255	916.35385	W	50-59	7	2
Pt 71	683.8965	691.3149	W	60-69	8	4
Pt 72	1188.09825	582.2856	W	50-59	9	5
Pt 73	550.87735	1172.97275	B	70-79	9	5
Pt 74	1099.11725	864.0709	W	70-79	9	5
Pt 75	594.8097	859.4658	W	60-69	7	2
Pt 76	793.06295	831.477	W	50-59	9	5
Pt 77	1073.53945	1499.36255	W	50-59	7	2
Pt 78	1147.6332	1997.42425	W	60-69	7	2
Pt 79	661.29665	734.7773	B	60-69	7	2
Pt 80	631.8921	690.11885	B	60-69	9	5
Pt 81	728.3864	1449.7911	W	50-59	9	5
Pt 82	959.1902	1157.422	W	60-69	7	3
Pt 83	427.62811	974.15515	B	60-69	7	3
Pt 84	477.80533	688.5239	W	60-69	7	2
Pt 85	768.39455	858.9097	W	50-59	6	1
Pt 86	840.13165	1070.13895	W	50-59	8	4
Pt 87	972.8153	906.9308	W	60-69	9	5
Pt 88	966.682	882.98605	W	50-59	7	2
Pt 89	1025.2124	1368.6096	B	60-69	7	2
Pt 90	946.59295	685.32055	B	50-59	7	3

Pt 91	753.1661	818.366	B	60-69	9	5
Pt 92	478.433725	548.70715	B	60-69	7	3
Pt 93	727.67165	1037.8135	B	<50	7	3
Pt 94	746.45235	414.86278	B	50-59	9	5
Pt 95	871.23535	1262.90845	B	70-79	7	2
Pt 96	1113.05535	751.75595	B	70-79	10	5
Pt 97	730.82485	557.73	W	50-59	6	1
Pt 98	657.58445	1288.768	W	60-69	7	2
Pt 99	1046.3156	1265.93455	B	60-69	7	3
Pt 100	1009.2841	901.8833	B	<50	9	5
Pt 101	664.32075	491.001365	B	<50	7	2
Pt 102	667.30015	704.07425	B	60-69	7	2
Pt 103	496.64528	1083.39595	B	50-59	7	2
Pt 104	567.0977	455.243935	B	50-59	9	5
Pt 105	530.91375	858.43445	B	50-59	9	5
Pt 106	507.5994	604.43585	B	60-69	7	2
Pt 107	448.92854	466.608695	B	50-59	7	2
Pt 108	612.9513	929.23315	B	60-69	9	5
Pt 109	553.49295	1607.2378	B	60-69	9	5
Pt 110	462.45237	737.031	B	60-69	9	5
Pt 111	611.32785	1061.9681	B	50-59	7	2
Pt 112	543.41735	439.44714	B	60-69	7	2
Pt 113	463.30368	833.37235	B	60-69	7	3

Pt 114	550.06905	909.19915	B	60-69	7	3
Pt 115	448.97843	765.3048	B	50-59	7	2
